# Development and validation of the first consensus gene-expression signature of operational tolerance in kidney transplantation, incorporating adjustment for immunosuppressive drug therapy

**DOI:** 10.1016/j.ebiom.2020.102899

**Published:** 2020-07-21

**Authors:** Sofia Christakoudi, Manohursingh Runglall, Paula Mobillo, Irene Rebollo-Mesa, Tjir-Li Tsui, Estefania Nova-Lamperti, Catharine Taube, Sonia Norris, Yogesh Kamra, Rachel Hilton, Titus Augustine, Sunil Bhandari, Richard Baker, David Berglund, Sue Carr, David Game, Sian Griffin, Philip A. Kalra, Robert Lewis, Patrick B. Mark, Stephen D. Marks, Iain MacPhee, William McKane, Markus G. Mohaupt, Estela Paz-Artal, Sui Phin Kon, Daniel Serón, Manish D. Sinha, Beatriz Tucker, Ondrej Viklický, Daniel Stahl, Robert I. Lechler, Graham M. Lord, Maria P. Hernandez-Fuentes

**Affiliations:** a MRC Centre for Transplantation, King's College London, Great Maze Pond, London SE1 9RT, UK; b Biostatistics and Health Informatics Department, Institute of Psychiatry, Psychology and Neuroscience, King's College London, 16 De Crespigny Park, London SE5 8AF, UK; c NIHR Biomedical Research Centre at Guy's & St Thomas’ NHS Foundation Trust and King's College London, Great Maze Pond, London SE1 9RT, UK; d Guy's and St Thomas’ NHS Foundation Trust, Great Maze Pond, London SE1 9RT, UK; e Manchester Royal Infirmary, Oxford Rd, Manchester M13 9WL, UK; f Hull University Teaching Hospitals NHS Trust, Anlaby Rd, Hull HU3 2JZ, UK; g St James's University Hospital, Beckett St, Leeds LS9 7TF, UK; h Department of Immunology, Genetics and Pathology, Uppsala University, Rudbecklaboratoriet, 751 85 Uppsala, Sweden; i Leicester General Hospital, Gwendolen Rd, Leicester LE5 4PW, UK; j Cardiff and Vale University Health Board, Cardiff CF14 4XW, UK; k Salford Royal NHS Foundation Trust, Stott Ln, Salford M6 8HD, UK; l Queen Alexandra Hospital, Southwick Hill Rd, Cosham, Portsmouth PO6 3LY, UK; m University of Glasgow, University Avenue, Glasgow G12 8QQ, UK; n Department of Paediatric Nephrology, Great Ormond Street Hospital for Children NHS Foundation Trust, Great Ormond Street, London WC1N 3JH, UK; o University College London Great Ormond Street Institute of Child Health, NIHR Great Ormond Street Hospital Biomedical Research Centre, London WC1N 1EH, UK; p St George's Hospital, Blackshaw Rd, London SW17 0QT, UK & Institute of Medical and Biomedical Education, St George's, University of London, Cranmer Terrace, London SW17 0RE; q Northern General Hospital, Herries Rd, Sheffield S5 7AU, UK; r Internal Medicine, Lindenhofgruppe Berne, Switzerland; s University of Bern, Berne, Switzerland; t School of Medicine, University of Nottingham, Nottingham NG5 1PB, UK; u Department of Immunology and imas12 Research Institute, University Hospital 12 de Octubre, Madrid, Spain; v King's College Hospital NHS Foundation Trust, Denmark Hill, London SE5 9RS, UK; w Hospital Universitario Vall d'Hebrón, Passeig de la Vall d'Hebron, 119-129, 08035 Barcelona, Spain; x Evelina London Children's Hospital, Westminster Bridge Rd, Lambeth, London SE1 7EH, UK; y King's Health Partners, Guy's Hospital, London SE1 9RT, UK; z Transplantační laboratoř, Institut klinické a experimentální medicíny (IKEM), Vídeňská 1958/9, 140 21 Praha 4, Czech Republic

**Keywords:** Kidney, Transplantation, Operational Tolerance, Biomarkers, Immunosuppressive Drugs, RT-qPCR, AP, anti-proliferative agent, AUC, area under the receiver operating characteristics curve, AZA, azathioprine, CNI, calcineurin inhibitor, CR, chronic rejection, CYC, cyclosporin, DSA, donor specific antibodies, eGFR, estimated glomerular filtration rate, GAMBIT, Genetic Analysis and Monitoring of Biomarkers of Immunological Tolerance study, HC, healthy control, HLA, human leucocyte antigens, IS, immunosuppression / immunosuppressive, KTR, kidney transplant recipient, MMF, mycophenolate-mofetil, mTOR, mammalian target of rapamycin, Non-TOL, non-tolerant, i.e. either a stable KTR or one with CR, OT, operational tolerance, PBMC, peripheral blood mononuclear cells, PRED, prednisolone, RNA, Ribonucleic acid, RT-qPCR, real time quantitative polymerase chain reaction, ST, stable, i.e. a KTR with stable kidney function, TAC, tacrolimus, TOL, tolerant, i.e. a KTR with established operational tolerance, TOL-positive, a KTR with predicted probability of tolerance above a defined cut-off

## Abstract

**Background:**

Kidney transplant recipients (KTRs) with “operational tolerance” (OT) maintain a functioning graft without immunosuppressive (IS) drugs, thus avoiding treatment complications. Nevertheless, IS drugs can influence gene-expression signatures aiming to identify OT among treated KTRs.

**Methods:**

We compared five published signatures of OT in peripheral blood samples from 18 tolerant, 183 stable, and 34 chronic rejector KTRs, using gene-expression levels with and without adjustment for IS drugs and regularised logistic regression.

**Findings:**

IS drugs explained up to 50% of the variability in gene-expression and 20–30% of the variability in the probability of OT predicted by signatures without drug adjustment. We present a parsimonious consensus gene-set to identify OT, derived from joint analysis of IS-drug-adjusted expression of five published signature gene-sets. This signature, including *CD40, CTLA4, HSD11B1, IGKV4–1, MZB1, NR3C2, and RAB40C* genes, showed an area under the curve 0⋅92 (95% confidence interval 0⋅88–0⋅94) in cross-validation and 0⋅97 (0⋅93–1⋅00) in six months follow-up samples.

**Interpretation:**

We advocate including adjustment for IS drug therapy in the development stage of gene-expression signatures of OT to reduce the risk of capturing features of treatment, which could be lost following IS drug minimisation or withdrawal. Our signature, however, would require further validation in an independent dataset and a biomarker-led trial.

**Funding:**

FP7-HEALTH-2012-INNOVATION-1 [305147:BIO-DrIM] (SC,IR-M,PM,DSt); MRC [G0801537/ID:88245] (MPH-F); MRC [MR/J006742/1] (IR-M); Guy's&StThomas’ Charity [R080530]&[R090782]; CONICYT-Bicentennial-Becas-Chile (EN-L); EU:FP7/2007–2013 [HEALTH-F5–2010–260687: The ONE Study] (MPH-F); Czech Ministry of Health [NV19–06–00031] (OV); NIHR-BRC Guy's&StThomas' NHS Foundation Trust and KCL (SC); UK Clinical Research Networks [portfolio:7521].

**Research in context**

**Evidence before this study**

A limited number of kidney transplant recipients (KTRs) can develop a state of “operational tolerance” (OT), in which they maintain their functioning graft after years of immunosuppressive (IS) drug withdrawal. Successfully identifying such patients remains a highly desirable clinical objective. This would allow for personalisation of therapy and reduction of the IS burden, thus avoiding the undesirable side effects of IS drugs, while maintaining graft function. Five gene-expression signatures have previously been published, which have all shown strong associations with OT. A major problem with identifying OT among treated stable KTRs, however, is that the features they share with untreated KTRs with established OT may be the result of IS drug exposure and could be lost once the drugs are changed, reduced, or withdrawn. Although some groups have acknowledged associations between their signatures and IS drugs, only our group, as far as we know, has used drug-adjusted gene-expression levels prior to applying a statistical algorithm for gene selection.

**Added value of this study**

We compared all five published signatures of OT using gene expression measured in peripheral blood samples from KTRs, collected in our previous signature development studies. We obtained gene-expression levels with real time quantitative polymerase chain reaction (RT-qPCR), which is a general analytical method widely available to clinical laboratories in and out of hospital environment, unlike microarray measurements. We showed that IS drugs could explain up to 32% of the variability observed in the predicted probability of OT based on signatures using unadjusted gene-expression levels and up to 50% of the variability in the expression of individual genes. Moreover, the predictive performance of signature gene-sets originally designed to use unadjusted gene expression deteriorated when a drug-adjustment step was introduced. To our knowledge, this is the first comparison of gene-expression signatures using adjustment for IS drugs and a “point of care” assay. Finally, we derived a consensus gene-set to identify OT in treated KTRs, by analysing IS-drug-adjusted expression levels for all published signature genes.

**Implications of all the available evidence**

When drug-adjustment is not performed at the signature development stage, the resultant signatures identify KTRs with gene-expression characteristics that are determined not only by OT, but sometimes by the pharmacological effects of the IS drug regimens they receive, or by other unknown reasons. Withdrawing IS drugs from KTRs dependent on their pharmacological effects would most likely put the survival of grafts at considerable risk. Our consensus signature validates genes previously identified in different datasets and uses drug-adjusted gene expression, thus minimising the risk of pharmacological influences. Nevertheless, a further validation in an independent external dataset would be required prior to a prospective biomarker-led clinical trial.

Alt-text: Unlabelled box

## 1 Introduction

Kidney transplantation increases survival of end-stage kidney disease but requires lifelong immunosuppression (IS) with one or a combination of IS drugs. Use of IS drugs, however, is associated with nephrotoxicity, metabolic changes, increased risk of type 2 diabetes mellitus, infections, and cancers. There is, therefore, a pressing need to safely minimise the dose and use of IS drugs without this leading to rejection.

Developing a status of “operational tolerance” (OT) is an attractive possibility, as it would enable transplant recipients to maintain a viable graft without the need for IS therapy, thus avoiding undesirable side effects. OT develops spontaneously and considerably more often in liver compared to kidney transplantation [1,2]. Nevertheless, there is a scientific interest in identifying those patients, among clinically stable kidney transplant recipients (KTRs), who have developed OT and in whom it would be appropriate to reduce or, potentially, completely withdraw IS drugs [3,4].

One approach to identifying patients with particular immunological characteristics is performing gene-expression analysis of peripheral blood, an easily obtainable biological sample amenable to standardisation, which avoids the risks of multiple biopsy sampling. Isolation of peripheral blood mononuclear cells (PBMC) is a laborious alternative and is more prone to laboratory variation. Consequently, our group and others have developed gene-expression signatures of OT in peripheral blood samples of KTRs. These signatures are based on statistical models that discriminate KTRs with already established OT, who have discontinued treatment for various reasons and lengths of time, from KTRs receiving IS drugs. However, a key point to consider when identifying treated KTRs as tolerant is knowing to what extent the features that they share with KTRs with already established OT without treatment are the result of IS drugs and whether their tolerance could be lost if the drugs were withdrawn. Immunosuppressive drugs are administered to maintain “pharmacological non-rejection”, which may, indeed, share molecular features with OT.

To account for the possible influence of IS drugs on gene expression in peripheral blood, we have used gene-expression levels adjusted for the most common IS drugs when developing signatures of OT and have argued that lack of adjustment can lead to confounding of gene-expression characteristics by IS drugs [5,6]. Although most groups have acknowledged the influence of drug regimens on gene-expression signatures of tolerance [7,8], they have not accounted for IS drug therapy during the signature development stage. To illustrate the impact that the lack of statistical adjustment of gene expression may have on the identification of potentially tolerant treated KTRs, we set to (1) compare our two signatures [6,9] with three published signatures of OT developed by other groups without statistical adjustment for drug therapy [10], [11], [12], and (2) to derive a parsimonious consensus gene-set among all five signatures using drug-adjusted gene-expression levels and real time quantitative polymerase chain reaction (RT-qPCR) analysis, an analytical method already applied in common validated clinical laboratory tests and, thus, amenable to translation into clinical practice [13].

## 2 Materials and methods

### 2.1 Patients and samples

Blood samples originated from KTRs recruited in the GAMBIT study (Genetic Analysis and Monitoring of Biomarkers of Immunological Tolerance), which were used and described previously [6]. Treated KTRs were either clinically stable or with chronic rejection (CR). A limited number of KTRs had established OT. Stable KTRs were over four years post-transplantation, with less than 15% change in the estimated glomerular filtration rate (eGFR) during the last 12 months. CR KTRs were over one year post-transplantation and diagnosed with immunologically driven chronic allograft nephropathy in graft biopsy (Banff 2007 or higher) in the last 12 months. KTRs with OT were more than 12 months off IS drugs, with less than 10% rise in serum creatinine since baseline. KTRs were recruited in 14 transplant centres. Samples were collected between September 2009 and December 2014. Healthy controls (HC) were also included for comparison.

For the purpose of this study, gene expression was measured in a total of 238 KTRs, providing two sets of samples. Time point one (T1-cohort) comprised samples from 18 tolerant (TOL), 186 stable, and 34 CR KTRs, and 12 HC. Time point two (T2-cohort) comprised follow-up samples collected approximately six months after the first sample from 70 of the KTRs in T1-cohort: 12 TOL, 43 stable, and 15 CR KTRs.

### 2.2 Ethics statement

Ethical approval was obtained from the National Research Ethics Service Committee London – Bloomsbury on 29 April 2009 (reference: 09/H0713/12). Written informed consent was obtained from all study participants.

### 2.3 Gene-expression analysis

Peripheral vein blood for gene-expression analysis was collected using Tempus™ Blood RNA Tubes (Life-Technologies). We measured gene-expression levels by RT-qPCR (Applied Biosystems), as previously described [6]. A list of full gene names and assay information is provided in Supplementary Table S1. Relative gene-expression values were calculated on log2 scale with the comparative –ΔCt method [14]. Table 1 specifies the five examined signature gene-sets and the house-keeping genes used as reference.

**Table 1:** Signature gene-sets.

Signature	Gene expression	Signature genes	House-keeping gene(s)	Ref
GAMBIT-g9	drug-adjusted	*ATXN3, BCL2A1, EEF1A1, GEMIN7A, IGLC1, MS4A4A, NFKBIA, RAB40C, TNFAIP3*	*HPRT*	[6]
GAMSTER-g4	drug-adjusted	*H6PD, HSD11B1, NR3C1, NR3C2*^a^	*HPRT*	[9]
ROEDDER-g3	unadjusted	*BNC2, CYP1B1, KLF6*	*HPRT*^b^	[12]
NEWELL-g2	unadjusted	*IGKV1D-13, IGKV4–1*^c^	*GAPDH*	[11]
DANGER-g6	unadjusted	*AKR1C3, CD40, CTLA4, ID3, MZB1, TCL1A*^d^	*ACTB, B2M, GAPDH, HPRT1*^e^	[10]
COMBINED-all	drug-adjusted	all the above genes with exclusions^f^	*HPRT*	–
COMBINED-g7	drug-adjusted	*CD40, CTLA4, HSD11B1, IGKV4–1, MZB1, NR3C2, RAB40C*	*HPRT*	–

a – *HSD11B2* from the original signature was excluded, as it was above the conventional threshold of 35Ct in 13% of the samples, i.e. it was not appropriate for routine real-time quantitative polymerase chain reaction (RT-qPCR) analysis;

b – we used *HPRT* because the original reference gene *S18* had very high levels compared to the other genes of interest;

c – although the published signature included three genes [11], the authors were unable to validate the *IGLL1* gene with RT-qPCR and we found a similarly unsatisfactory analytical performance for this gene in the Fluidigm platform [6];

d – in the original signature the six genes were included in a composite score, together with two age parameters, which we did not consider in the current analysis for comparability with other signatures and because the enhancement of group discrimination by risk factors would be applicable to all signatures;

e – the geometric mean of the four genes was used and *HPRT1* was analysed with a different assay than *HPRT*, as per the original signature;

f – a signature including all genes, but with elastic net penalty favouring gene exclusion (alpha=0⋅95), such that the median regression coefficients from 600 models (100 repeats of six-fold cross-validation cycles) are non-zero for 14 genes; **Ref** – reference to the published original signature; Full gene names are listed in Supplementary Table S1.

### 2.4 Calibration of published signatures

The term “signature” refers to a multivariable statistical model based on gene-expression values. In this study we calibrated the published signatures, i.e. determined the elastic net regression coefficients, using RT-qPCR gene-expression values for T1-cohort. We trained the statistical models to discriminate KTRs with established OT “off treatment” (TOL) from the joint group of stable and CR KTRs receiving treatment (Non-TOL). We calibrated and compared two versions of each signature: using unadjusted and using drug-adjusted gene-expression values.

For drug adjustment, we used multivariable linear regression models in samples from Non-TOL KTRs from T1-cohort. The outcome variable in each drug adjustment model was gene expression (–ΔCt*_GENE_*) and the exposure variables were indicators of drug therapy defined as follows: off or on prednisolone (PRED); off a calcineurin inhibitor (CNI), or on cyclosporine (CYC), or on tacrolimus (TAC); off an antiproliferative agent (AP), or on azathioprine (AZA), or on mycophenolate mofetil (MMF). The equation of the drug-adjustment model was: –ΔCt*_GENE_* ~ PRED + CNI + AP [6]. We calculated drug-adjusted gene-expression values for all KTRs, including the T2-cohort and HCs, as the residuals of the drug-adjustment models, i.e. as the difference between the observed value of –ΔCt*_GENE_* and the value predicted from the drug adjustment model. These residuals capture the variability in gene expression not explained by IS drugs. The drug therapy indicators for TOL patients and HCs were set to “off treatment”. The version of each signature (with or without drug adjustment) used in the original publication (Table 1) is referred to as “original”.

We trained the gene-expression signatures using multivariable regularised logistic regression with elastic net penalty [6,9]. This includes a mixture of two penalties: ridge, which preserves all genes in the model, and lasso, which forces gene exclusion by vigorous shrinkage of the regression coefficients to zero and selects only one gene among a set of dependent/correlated genes which is most informative for the discrimination between TOL and Non-TOL KTRs (package “glmnet”) [15]. We set the parameter defining the proportion of ridge and lasso close to ridge regression (alpha=0⋅05), in order to retain the pre-selected sets of genes in the models, even if they were dependent/correlated, but also to improve model optimisation by permitting exclusion of genes with completely negligible contribution to OT discrimination. We optimised the second penalty parameter (lambda) as the median of 100 repeats of six-fold cross-validation cycles incorporated within function “cv.glmnet”*.*

We derived the regression coefficients for each “final” signature model as the median of all corresponding values from the models generated during the cross-validation cycles described in section “Validation strategy”.

### 2.5 Development of a consensus signature

To determine the most informative genes for OT discrimination after accounting for IS regimens, we used samples from T1-cohort and included all genes from the five examined signature gene-sets in one model (COMBINED-all). We performed statistical gene selection by setting the penalty parameter close to lasso (alpha=0⋅95). We included in the parsimonious consensus signature those genes which were preserved (i.e. had a non-zero regression coefficient) in at least 75% of all elastic net models generated in a set of cross-validation cycles described in section “Validation strategy”. We defined the drug-adjustment models and the regression coefficients for the final consensus signature model as described in the previous section.

### 2.6 Validation strategy

First, we performed a six-fold cross-validation in T1-cohort, in order to reduce the risk of overfitting. Each cross-validation cycle included splitting at random (in strata by clinical type) the samples from T1-cohort into a training and a test subset. All steps of signature development or calibration were performed in the training subset. This included the definition of the drug-adjustment models and the estimation of the elastic net regression coefficients, using in each model the complete gene-set for a given signature, as listed in Table 1. The model based on the training subset was used to predict the probability of tolerance for the left-out samples from the test subset, which had remained “unseen” during the model training phase. Thus, each cross-validation cycle, comprising six model training repeats, generated a single predicted probability of tolerance for each patient. We repeated the cross-validation cycle 100 times in order to reduce the influence of extreme random splits generated by chance. This generated 100 sets of predicted probabilities of OT for T1-cohort, 600 drug-adjustment models for each gene, and 600 elastic net models for each examined signature. The medians of the regression coefficients of these cross-validation models were used in the final model for each signature. A step-by-step explanation of the cross-validation algorithm is included in Supplementary Methods.

Second, we used the samples in T2-cohort as a longitudinal validation dataset. We used the final “model” for each signature to derive the probability of OT for T2-cohort, as well as for HCs.

### 2.7 Statistical evaluation and comparisons

We used a Wilcoxon-Mann-Witney test for pairwise group comparisons not involving IS drugs. We compared groups of patients on and off a given IS drug using linear regression models based on samples from Non-TOL KTRs in T1-cohort and including adjustments for all other examined IS drugs. For genes and signatures showing statistically significant associations with IS drugs, we additionally examined dose effect, replacing in the above linear regression models the categorical variable for the corresponding IS drug with a continuous variable for dose and retaining the adjustment for all other examined drugs. We excluded KTRs receiving CYC for models examining the dose of TAC and *vice versa*, as these drugs may have comparable influence on gene expression, and similarly excluded KTRs receiving AZA from models examining the dose of MMF and *vice versa*. Models examining associations of signatures with IS drugs included as an outcome variable the probability of OT derived from the final model for each signature transformed to log-odds with log(probability/(1-probability)). This transformation enabled conversion from the probability scale (restricted between 0 and 1) to a continuous scale required in linear regression.

We evaluated the influence of individual IS drugs with the percentage of explained variability (R^2^). For gene-expression values, R^2^ originated from the corresponding 600 drug-adjustment models created during the cross-validation cycles. For the probability of OT, R^2^ originated from a linear regression model in T1-cohort, including as explanatory variables indicators of drug therapy and as outcome variable the probability of OT transformed to log-odds, as described above.

We used the area under the receiver operating characteristics (ROC) curve (AUC) with a 95% DeLong confidence interval to evaluate signature performance, i.e. OT discrimination (package “pROC”) [16]. We compared the AUC of the unadjusted and the drug-adjusted variants of each signature with DeLong's test for paired ROC curves. We calculated sensitivity and specificity using as a conservative uniform cut-off for all signatures the median of the predicted probabilities of OT for the group of TOL KTRs. Other groups have used more lenient cut-offs to define TOL-positivity, e.g. the lowest level of gene expression in TOL KTRs [17]. We compared the identification of TOL-positivity by two signatures with Cohen's kappa index for interrater agreement, with kappa=1 indicating complete agreement and kappa=0 indicating complete lack of agreement (package “irr”) [18].

We summarised the regression coefficients and all statistical indices derived for the repeats of the cross-validation cycles with the median and the 2⋅5^th^–97⋅5^th^ centile range, and for summaries of elastic net regression coefficients, also the 25^th^−75^th^ centile range.

To avoid leverage of extreme values on regression coefficients and statistical test, we recoded outliers in gene expression to the next highest or lowest value in all models and excluded from dose response models KTRs with doses of IS drugs in the top 2⋅5 centiles. We imputed missing gene-expression values with the k-nearest neighbour algorithm from package “impute” [19]. In this study missingness was negligible, with missing only two out of some 9000 gene expression values.

We performed all statistical analyses in R version 3.2.2 [20].

### 2.8 Data sharing

We have made data supporting this study available within the main and supplementary sections of our manuscript. According to UK research councils’ common principles on Data Policy, any further data related to this study will be available through application to the Biobank “Transplantation, Immunology and Nephrology Tissue and Information Nexus” (TIN-TIN) based at King's College London, London UK. Ethical application reviewed and approved by London - Bromley Research Ethics Committee in September 2019 Ref: 17/LO/0220. Applications should be directed to Dr Paramit Chowdhury, Head of the Biobank. Guys and St Thomas’ NHS Foundation Trust. Renal Unit, 6th floor Borough Wing. Guy's Hospital. Great Maze Pond. London SE1 9RT. Paramit.Chowdhury@gstt.nhs.uk

## 3 Results

The demographic characteristics of all study participants and the IS drugs they received are summarised in Table 2.

**Table 2:** Demographic characteristics and immunosuppressive drug treatment of study participants.

Cohort	T1-cohort (total)			T1-cohort (T2 match subset)			T2-cohort		
Patient type	CR	Stable	TOL	CR	Stable	TOL	CR	Stable	TOL
Number	34	186	18	15	43	12	15	43	12
T1-T2 time^a^	–	–	–	–	–	–	6⋅4 (3⋅0)	7⋅1 (3⋅2)	5⋅4 (2⋅3)
Age^a^	44⋅7 (14⋅4)	50⋅7 (13⋅2)	48⋅5 (14⋅2)	44⋅3 (14⋅7)	48⋅9 (13⋅0)	50⋅3 (15⋅1)	44⋅8 (14⋅5)	49⋅4 (13⋅1)	50⋅9 (14⋅9)
Time from Tx^a^	9⋅5 (7⋅1)	14⋅8 (7⋅9)	19⋅0 (8⋅1)	6⋅3 (4⋅0)	15⋅1 (6⋅9)	20⋅8 (8⋅7)	6⋅8 (4⋅0)	15⋅6 (6⋅9)	21⋅2 (8⋅6)
eGFR^a^	33⋅1 (12⋅7)	63⋅9 (22⋅9)	60⋅4 (15⋅7)	33⋅3 (11⋅4)	61⋅3 (25⋅7)	61⋅7 (12⋅5)	32⋅2 (10⋅3)	58⋅6 (20⋅1)	66⋅8 (21⋅1)
Female^b^	11 (32⋅4)	60 (32⋅3)	4 (22⋅2)	4 (26⋅7)	12 (27⋅9)	2 (16⋅7)	4 (26⋅7)	12 (27⋅9)	2 (16⋅7)
Ethnicity^b^									
White	28 (82⋅4)	162 (87⋅1)	16 (88⋅9)	11 (73⋅3)	38 (88⋅4)	11 (91⋅7)	11 (73⋅3)	38 (88⋅4)	11 (91⋅7)
Asian	1 (2⋅9)	6 (3⋅2)	–	–	2 (4⋅7)	–	–	2 (4⋅7)	–
Black	3 (8⋅8)	8 (4⋅3)	–	2 (13⋅3)	2 (4⋅7)	–	2 (13⋅3)	2 (4⋅7)	–
Other / unknown	2 (5⋅9)	10 (5⋅4)	2 (11⋅1)	2 (13⋅3)	1 (2⋅3)	1 (8⋅3)	2 (13⋅3)	1 (2⋅3)	1 (8⋅3)
Living donor^b^	12 (35⋅3)	57 (30⋅6)	8 (44⋅4)	6 (40⋅0)	11 (25⋅6)	7 (58⋅3)	6 (40⋅0)	11 (25⋅6)	7 (58⋅3)
DSA^b^	15 (44⋅1)	16 (8⋅6)	3 (16⋅7)	6 (40⋅0)	5 (11⋅6)	2 (16⋅7)	6 (40⋅0)	5 (11⋅6)	2 (16⋅7)
HLA mismatch^b^									
None	–	18 (9⋅7)	5 (27⋅8)	–	2 (4⋅7)	3 (25⋅0)	–	2 (4⋅7)	3 (25⋅0)
HLA A only	–	8 (4⋅3)	1 (5⋅6)	–	2 (4⋅7)	1 (8⋅3)	–	2 (4⋅7)	1 (8⋅3)
HLA B only	2 (5⋅9)	14 (7⋅5)	–	–	3 (7⋅0)	–	–	3 (7⋅0)	–
HLA DR only	1 (2⋅9)	1 (0⋅5)	–	1 (6⋅7)	1 (2⋅3)	–	1 (6⋅7)	1 (2⋅3)	–
HLA A and B	8 (23⋅5)	39 (21⋅0)	2 (11⋅1)	6 (40⋅0)	6 (14⋅0)	1 (8⋅3)	6 (40⋅0)	6 (14⋅0)	1 (8⋅3)
HLA A and DR	3 (8⋅8)	12 (6⋅5)	–	1 (6⋅7)	5 (11⋅6)	–	1 (6⋅7)	5 (11⋅6)	–
HLA B and DR	3 (8⋅8)	13 (7⋅0)	–	2 (13⋅3)	5 (11⋅6)	–	2 (13⋅3)	5 (11⋅6)	–
HLA A, B and DR	14 (41⋅2)	60 (32⋅3)	7 (38⋅9)	4 (26⋅7)	11 (25⋅6)	5 (41⋅7)	4 (26⋅7)	11 (25⋅6)	5 (41⋅7)
Unknown	3 (8⋅8)	21 (11⋅3)	3 (16⋅7)	1 (6⋅7)	8 (18⋅6)	2 (16⋅7)	1 (6⋅7)	8 (18⋅6)	2 (16⋅7)
IS drugs^b^									
On PRED	24 (70⋅6)	78 (41⋅9)	–	10 (66⋅7)	23 (53⋅5)	–	11 (73⋅3)	23 (53⋅5)	–
Off CNI	2 (5⋅9)	34 (18⋅3)	18 (100)	1 (6⋅7)	7 (16⋅3)	12 (100)	1 (6⋅7)	7 (16⋅3)	12 (100)
On CYC	4 (11⋅8)	96 (51⋅6)	–	1 (6⋅7)	25 (58⋅1)	–	1 (6⋅7)	25 (58⋅1)	–
On TAC	28 (82⋅4)	56 (30⋅1)	–	13 (86⋅7)	11 (25⋅6)	–	13 (86⋅7)	11 (25⋅6)	–
Off AP	6 (17⋅6)	33 (17⋅7)	18 (100)	3 (20⋅0)	11 (25⋅6)	12 (100)	2 (13⋅3)	11 (25⋅6)	12 (100)
On AZA	5 (14⋅7)	67 (36⋅0)	–	3 (20⋅0)	15 (34⋅9)	–	3 (20⋅0)	15 (34⋅9)	–
On MMF	23 (67⋅6)	86 (46⋅2)	–	9 (60⋅0)	17 (39⋅5)	–	10 (66⋅7)	17 (39⋅5)	–
IS drug doses^c^									
PRED	5⋅0 (3⋅1)	5⋅0 (0)	–	6⋅9 (5⋅0)	5⋅0 (1⋅0)	–	7⋅5 (5⋅0)	5⋅0 (2⋅2)	–
CYC	150 (19)	150 (100)	–	125 (0)	125 (125)	–	200 (0)	150 (100)	–
TAC	4⋅5 (2⋅1)	3⋅8 (3⋅0)	–	5⋅0 (6⋅0)	4⋅0 (1⋅5)	–	5⋅0 (4⋅5)	4⋅0 (2⋅0)	–
AZA	100 (75)	100 (50)	–	150 (25)	75 (62)	–	150 (25)	75 (62)	–
MMF	1000 (750)	1000 (500)	–	1037 (1000)	1000 (500)	–	509 (1390)	1000 (500)	–

a – summarised with mean (standard deviation);

b – summarised with number (percentage from total in group);

c – summarised with median (interquartile range) for patients receiving the corresponding drug; **Age** – age at sample collection (years); **AP** – anti-proliferative; **AZA** – azathioprine; **CNI** – calcineurin inhibitor; **CYC** – cyclosporin; **CR** – chronic rejector kidney transplant recipients (KTRs); **DSA** – donor specific antibodies; **eGFR** – estimated glomerular filtration rate; **HLA** – human leucocyte antigens; **IS** – immunosuppressive; **KTRs** – kidney transplant recipients; **MMF** – mycophenolate-mofetil; **PRED** – prednisolone; **TAC** – tacrolimus; **TOL** – KTRs with operational tolerance; **T1-T2 time** – time between timepoints 1 and 2 (months); **Time from Tx** – time from transplantation (years); **T1-cohort** – participants at baseline; **T2-cohort** – participants from T1-cohort with a follow-up sample; **T1-cohort (T2 match subset)** – the subset of the cohort at time point one that included only the patients providing samples in both time points; Two of the 12 healthy controls were women and the mean age at sample collection was 49•3 (standard deviation=12•8) years. eGFR was available for 12 TOL, 173 stable and 31 CR KTRs from T1-cohort and for 10 TOL, 40 stable and 14 CR KTRs from T2-cohort. KTRs were recruited in 14 transplant centres and samples were collected between September 2009 and December 2014 [6].

### 3.1 Immunosuppressive drugs influence relevant gene-expression levels

IS drug therapy affected the expression of individual genes from all signature gene-sets (Supplementary Fig. S1). IS drugs explained as much as 50% of the variability observed in the expression of the *TCL1A* gene from DANGER-g6, and some 20–30% for the *IGLC1* and *NFKBIA* genes from GAMBIT-g9, the *BNC2* gene from ROEDDER-g3, both *IGKV1D-13* and *IGKV4–1* genes from NEWELL-g2, and the *CD40, ID3,* and *MZB1* genes from DANGER-g6.

PRED affected most genes. With PRED therapy, expression was lower for all genes from NEWELL-g2 and DANGER-g6, the *HSD11B1* and *NR3C2* genes from GAMSTER-g4, and the *EEF1A1* and *IGLC1* genes from GAMBIT-g9. However, expression was higher with PRED for the *BCL2A1* and *NFKBIA* genes from *GAMBIT-g9,* the *H6PD* gene from GAMSTER-g4, and the *KLF6* gene from ROEDDER-g3 (Supplementary Fig. S1). With CNI therapy, expression was higher for the *IGLC1* gene from GAMBIT-g9, both *IGKV1D-13* and *IGKV4–1* genes from NEWELL-g2, and the *ID3* gene from DANGER-g6, for CYC and TAC alike. With AZA therapy, expression was lower for the *BNC2* gene from ROEDDER-g3 and the *AKR1C3, CD40,* and *TCL1A* genes from DANGER-g6. With MMF therapy, expression was lower for both *IGKV1D-13* and *IGKV4–1* genes from NEWELL-g2 and the *MZB1* gene from DANGER-g6 (Supplementary Fig. S1). Although the variability of doses was limited for PRED (63% on 5 mg/day) and TAC (77% on 2–6 mg/day), dose response associations were observed between the genes and IS drugs highlighted above (Supplementary Fig. S2). Dose response associations were more robust for CYC, AZA and MMF, which had wider ranging doses.

**Fig. 1:**
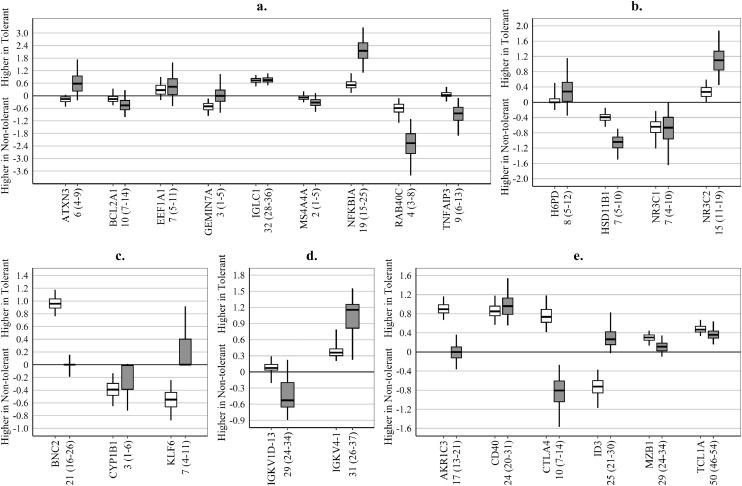
Influence of drug-adjustment of gene-expression values on the regression coefficients for individual genes included in the examined signatures. (**a**) – **GAMBIT-g9**; (**b**) – **GAMSTER-g4**; (**c**) – **ROEDDER-g3**; (**d**) – **NEWELL-g2**; (**e**) – **DANGER-g6; Regression coefficients** – the larger the absolute value, the bigger the contribution of the corresponding gene to the signature model, i.e. genes with regression coefficients close to zero had minimal or no contribution to the discrimination of operational tolerance; **Box and whiskers** – summary of regression coefficients from the individual elastic net models (penalty parameter alpha=0⋅05) in 100 repeats of six-fold cross-validation cycles (600 models in total) – horizontal line: median, box – 25^th^–75^th^ centile range; whiskers – 2⋅5^th^–97⋅5^th^ centile range; **White boxes** – summary of regression coefficients from models based on unadjusted gene-expression values, derived with the –ΔCt method, relative to *HPRT* as a house-keeping gene for GAMBIT-g9, GAMSTER-g4 and ROEDDER-g3, *GAPDH* for NEWELL-g2 and the geometric mean of *ACTB, B2M, GAPDH* and *HPRT1* for DANGER-g6 (gene details are included in Supplementary Table S1); **Grey boxes** – summary of regression coefficients from models based on drug-adjusted gene expression values, derived as the residuals from linear models regressing gene-expression values for each gene on drug therapy (prednisolone (PRED) – on/off, calcineurin inhibitors (CNI) – off, or on cyclosporine (CYC), or on tacrolimus (TAC), anti-proliferative agent (AP) – off, or on azathioprine (AZA), or on mycophenolate mofetil (MMF); **Numbers (x-axis)** – summary of the percentage of variability explained by drugs in the drug-adjustment models of the cross-validation cycles: median (2⋅5^th^-97⋅5^th^ centile range); Signature gene-sets are described in Table 1.

Adjustment of gene-expression values for IS drug therapy altered the contribution of some genes to the signatures of OT, but not proportionally to the variability in gene-expression explained by IS drugs (Fig. 1). For example, after drug adjustment the *IGCL1, IGKV4−1, CD40,* and *TCLA1* genes, all with R^2^>20%, retained their contribution to the discrimination of TOL from Non-TOL KTRs (Fig. 1a,d,e). At the same time, the *AKR1C3* and *BCN2* genes, also with R^2^≈20%, completely lost discriminating ability, as the median of their regression coefficients became zero (Fig. 1c,e), while the regression coefficient completely changed sign for the *ID3* gene, also with R^2^≈25% (Fig. 1e).

### 3.2 Immunosuppressive drugs influence the predicted probability of tolerance

Drug-adjustment affected, to some extent, the discrimination between TOL and Non-TOL KTRs for all signatures (Supplementary Fig. S3). Notably, in T1-cohort, the AUC of the drug-adjusted version was considerably higher than the unadjusted version for GAMSTER-g4 (Table 3). However, the drug-adjusted version for ROEDDER-g3 effectively lost OT discrimination and the drug-adjusted version of DANGER-g6 had lower AUC and poor agreement of the identification of TOL-positivity when compared to the unadjusted version. The number of Non-TOL KTRs identified as TOL-positive differed between signatures and between the drug-adjusted and the unadjusted version of each signature.

**Table 3:** Comparison of the predictive performance of signatures calibrated with unadjusted and drug-adjusted gene-expression values.

Signature		Cross-validation		T1-cohort				
	Drug adj.	AUC	Specificity	AUC	*p*	Spec	TOL-posST/CR	Cohen'skappa
GAMBIT-g9	no	0⋅76 (0⋅72–0⋅80)	0⋅84 (0⋅79–0⋅89)	0⋅86 (0⋅77–0⋅94)	0⋅12	0⋅94	11/3	0⋅55
	yes	0⋅82 (0⋅78–0⋅86)	0⋅91 (0⋅86–0⋅95)	0⋅90 (0⋅83–0⋅96)		0⋅96	6/3	
GAMSTER-g4	no	0⋅62 (0⋅56–0⋅66)	0⋅71 (0⋅60–0⋅76)	0⋅70 (0⋅58–0⋅82)	0⋅0024	0⋅79	41/6	0⋅54
	yes	0⋅80 (0⋅77–0⋅82)	0⋅87 (0⋅81–0⋅92)	0⋅83 (0⋅75–0⋅91)		0⋅90	18/4	
ROEDDER-g3	no	0⋅76 (0⋅74–0⋅78)	0⋅80 (0⋅76–0⋅83)	0⋅79 (0⋅72–0⋅87)	0⋅00033	0⋅82	35/4	0⋅10
	yes	0⋅53 (0⋅43–0⋅60)	0⋅44 (0⋅23–0⋅63)	0⋅58 (0⋅44–0⋅72)		0⋅58	75/18	
NEWELL-g2	no	0⋅72 (0⋅69–0⋅73)	0⋅79 (0⋅76–0⋅83)	0⋅75 (0⋅62–0⋅87)	0⋅19	0⋅85	30/4	0⋅54
	yes	0⋅75 (0⋅72–0⋅77)	0⋅86 (0⋅81–0⋅90)	0⋅77 (0⋅65–0⋅89)		0⋅87	25/4	
DANGER-g6	no	0⋅85 (0⋅84–0⋅86)	0⋅92 (0⋅90–0⋅94)	0⋅89 (0.81–0⋅96)	0⋅029	0⋅95	10/1	0⋅29
	yes	0⋅76 (0⋅74–0⋅78)	0⋅90 (0⋅85–0⋅92)	0⋅•79 (0⋅67–0⋅92)		0⋅90	17/4	
COMBINED-all	yes	0⋅89 (0⋅84–0⋅92)	0⋅95 (0⋅92–0⋅97)	0⋅97 (0⋅94–0⋅99)	0⋅066	0⋅98	3/2	0⋅92
COMBINED-g7	yes	0⋅92 (0⋅88–0⋅94)	0⋅96 (0⋅94–0⋅98)	0⋅96 (0⋅93–0⋅99)		0⋅97	5/1	0⋅88

**COMBINED-all** – a signature including all genes from the five examined signature gene-sets with elastic net penalty alpha=0⋅95, enabling gene exclusion (signature gene-sets are listed in Table 1); **Drug Adj** – indicates whether drug adjustment of the gene-expression values was used; **AUC** – area under the receiver operation characteristics (ROC) curve; **Cross-validation** – summaries from 100 repeats of six-fold cross-validation cycles: median (2⋅5^th^–97⋅5^th^ centile range); **T1-cohort** – performance of the final model (95% DeLong confidence interval for AUC); **Specificity (Spec)** – determined with a cut-off at the median predicted probability of tolerance among patients with operational tolerance at every cross-validation cycle, ensuring 50% sensitivity for all signatures; ***p*** – *p*-value from DeLong's test for comparison of the AUC of paired ROC curves for the drug-adjusted vs. unadjusted versions of each signature, or the parsimonious seven-genes vs the all-genes COMBINED signature; **TOL-pos** – stable (ST, out of 186) / chronic rejector (CR, out of 34) patients with predicted probability of tolerance higher than the cut-off described above, i.e. identified as TOL-positive; **Cohen's kappa** –index of interrater agreement, comparing identification of TOL-positivity by the drug-adjusted and unadjusted version of each signature, or the two COMBINED signatures, with kappa=1 indicating complete agreement and kappa=0 indicating complete lack of agreement.

The AUCs of all signatures in T2-cohort were comparable to T1-cohort, but the agreement in the identification of TOL-positivity between T2-cohort and T1-cohort was considerably better for the drug-adjusted version of GAMSTER-g4 and DANGER-g6 (kappa≈0⋅75) compared to the unadjusted version (kappa≈0⋅35) (Table 4). There was no evidence that any signature preferentially classified KTRs with higher eGFR as TOL-positive (Supplementary Fig. S4).

**Table 4:** Comparison of the predictive performance of signatures in T1-cohort (development/calibration) and T2-cohort (longitudinal validation).

	Drug	Prob	T1-cohort (T2 match subset)			T2-cohort			Cohen's
Signature	Adj.	Cut-off	AUC	Sens	Spec	AUC	Sens	Spec	kappa
GAMBIT-g9	no	0⋅13	0⋅89 (0⋅79–0⋅98)	0⋅50	0⋅95	0⋅83 (0⋅73–0⋅94)	0⋅42	0⋅90	0⋅53
	yes	0⋅20	0⋅89 (0⋅80–0⋅98)	0⋅58	0⋅95	0⋅84 (0⋅73–0⋅96)	0⋅50	0⋅86	0⋅50
GAMSTER-g4	no	0⋅09	0⋅68 (0⋅52–0⋅84)	0⋅42	0⋅74	0⋅69 (0⋅53–0⋅84)	0⋅33	0⋅93	0⋅32
	yes	0⋅14	0⋅81 (0⋅69–0⋅92)	0⋅50	0⋅88	0⋅83 (0⋅74–0⋅93)	0⋅42	0⋅91	0⋅74
ROEDDER-g3	no	0⋅12	0⋅87 (0⋅79–0⋅96)	0⋅50	0⋅90	0⋅83 (0⋅73–0⋅93)	0⋅42	0⋅90	0⋅64
	yes	0⋅08	0⋅57 (0⋅40–0⋅75)	0⋅67	0⋅50	0⋅52 (0⋅35–0⋅69)	0⋅67	0⋅41	0⋅39
NEWELL-g2	no	0⋅12	0⋅83 (0⋅71–0⋅96)	0⋅58	0⋅90	0⋅82 (0⋅69–0⋅94)	0⋅42	0⋅88	0⋅46
	yes	0⋅12	0⋅85 (0⋅71–0⋅98)	0⋅67	0⋅91	0⋅84 (0⋅72–0⋅96)	0⋅50	0⋅84	0⋅46
DANGER-g6	no	0⋅24	0⋅97 (0⋅92–1⋅00)	0⋅58	1⋅00	0⋅93 (0⋅87–0⋅99)	0⋅50	0⋅98	0⋅37
	yes	0⋅15	0⋅86 (0⋅72–0⋅99)	0⋅58	0⋅91	0⋅91 (0⋅83–0⋅98)	0⋅58	0⋅95	0⋅78
COMBINED-all	yes	0⋅30	0⋅95 (0⋅90–1⋅00)	0⋅58	0⋅95	0⋅97 (0⋅94–1⋅00)	0⋅67	0⋅97	0⋅65
COMBINED-g7	yes	0⋅32	0⋅95 (0⋅90–1⋅00)	0⋅58	0⋅97	0⋅97 (0⋅93–1⋅00)	0⋅83	0⋅98	0⋅65

**COMBINED-all** – a signature including all genes from the five examined signature gene-sets with elastic net penalty alpha=0⋅95, enabling gene exclusion (signature gene-sets are listed in Table 1); **Drug Adj**. – indicates whether drug adjustment of the gene-expression values was used; **AUC** – area under the receiver operation characteristics (ROC) curve (95% DeLong confidence interval); **T1-cohort** (T2 match subset) – the subset of the cohort at time point one that included only the patients providing samples at both time points; **T2-cohort** – 70 patients from T1-cohort providing follow-up samples at time point two (this was used as a longitudinal validation set); **Prob Cut-off** – probability cut-off used to calculate specificity and sensitivity, determined as the median predicted probability of tolerance among all patients with operational tolerance in the complete T1-cohort, i.e. accounts for 50% sensitivity in the total T1-cohort; **Sens / Spec** – sensitivity and specificity; **Cohen's kappa** –index of interrater agreement, comparing identification of TOL-positivity in T1-cohort and in T2-cohort.

**Fig. 2:**
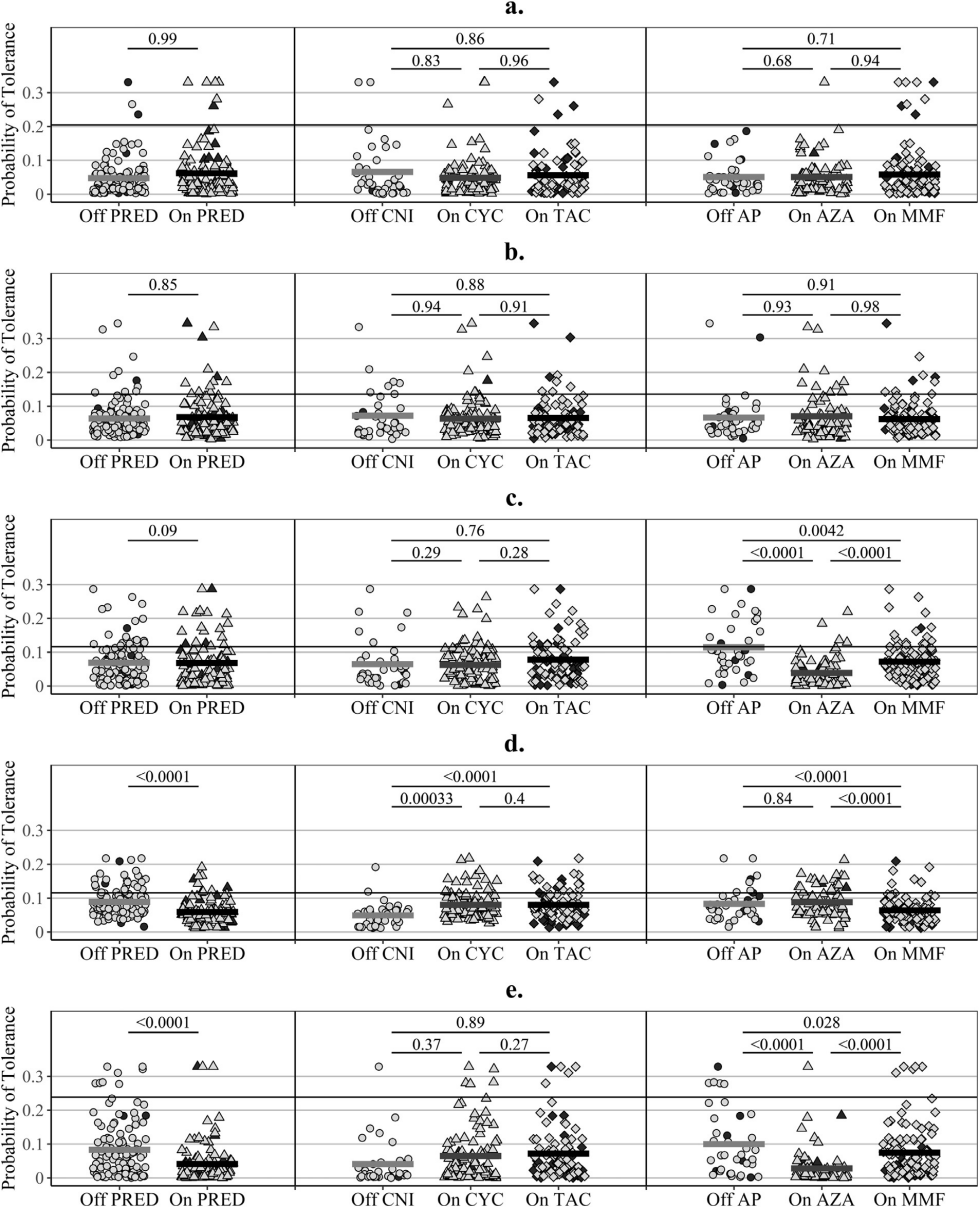
Influence of immunosuppressive drugs on the predicted probabilities of tolerance. (**a**) – **GAMBIT-g9** (R^2^≤1%); (**b**) – **GAMBIT-g4** (R^2^≤1%); (**c**) – **ROEDDER-g3** (R^2^=21%); (**d**) – **NEWELL-g2** (R^2^=31%); (**e**) – **DANDER-g6** (R^2^=33%); **R**^**2**^ – percentage of explained variability from linear models regressing the predicted probability of tolerance (log-odds) on immunosuppressive (IS) drugs, i.e. the percentage of variability in the probabilities, which is explained by IS drugs coded as follows: prednisolone (PRED) – off or on; calcineurin inhibitors (CNI) – off, on cyclosporine (CYC), or on tacrolimus (TAC); anti-proliferative agents (AP) – off, on azathioprine (AZA), or on mycophenolate mofetil (MMF) (log-odds convert the probability scale, restricted between zero and one, to a continuous scale required for linear regression); ***p*****-values** – derived from Wald tests in the linear regression models described above, i.e. adjusted for therapy with other IS drugs (of note, most patients off CNI received prednisolone and *vice versa*, which would explain why differences in gene-expression levels observed between off/on CNI are not always reflected in the adjusted p-values); **pail symbols** – stable kidney transplant recipients; **dark symbols** – patients with chronic rejection (CR); **horizontal lines per group** – mean probability of tolerance for the group; **horizontal reference lines** – median probability of tolerance in tolerant patients, i.e. a cut-off ensuring 50% sensitivity.

The predicted probabilities of tolerance from the original version of GAMBIT-g9 and GAMSTER-g4, using drug-adjusted gene-expression levels, were not influenced by IS drugs (Fig. 2a,b). On the contrary, IS drugs explained 20−30% of the variability observed in the probabilities of tolerance predicted with the original version of ROEDDER-g3, NEWELL-g2, and DANGER-g6, using unadjusted gene-expression levels (Fig. 2c−e). The predicted probabilities of tolerance were affected by individual IS drugs as follows: lower with AP therapy, especially with AZA, for ROEDDER-g3 (Fig. 2c); lower with PRED and MMF therapy, and higher with CNI therapy for NEWELL-g2 (Fig. 2d); and lower with PRED and AZA therapy for DANGER-g6 (Fig. 2e), with some dose effect (Supplementary Fig. S5).

### 3.3 Development of the consensus drug-adjusted gene-expression signature

**Fig. 3:**
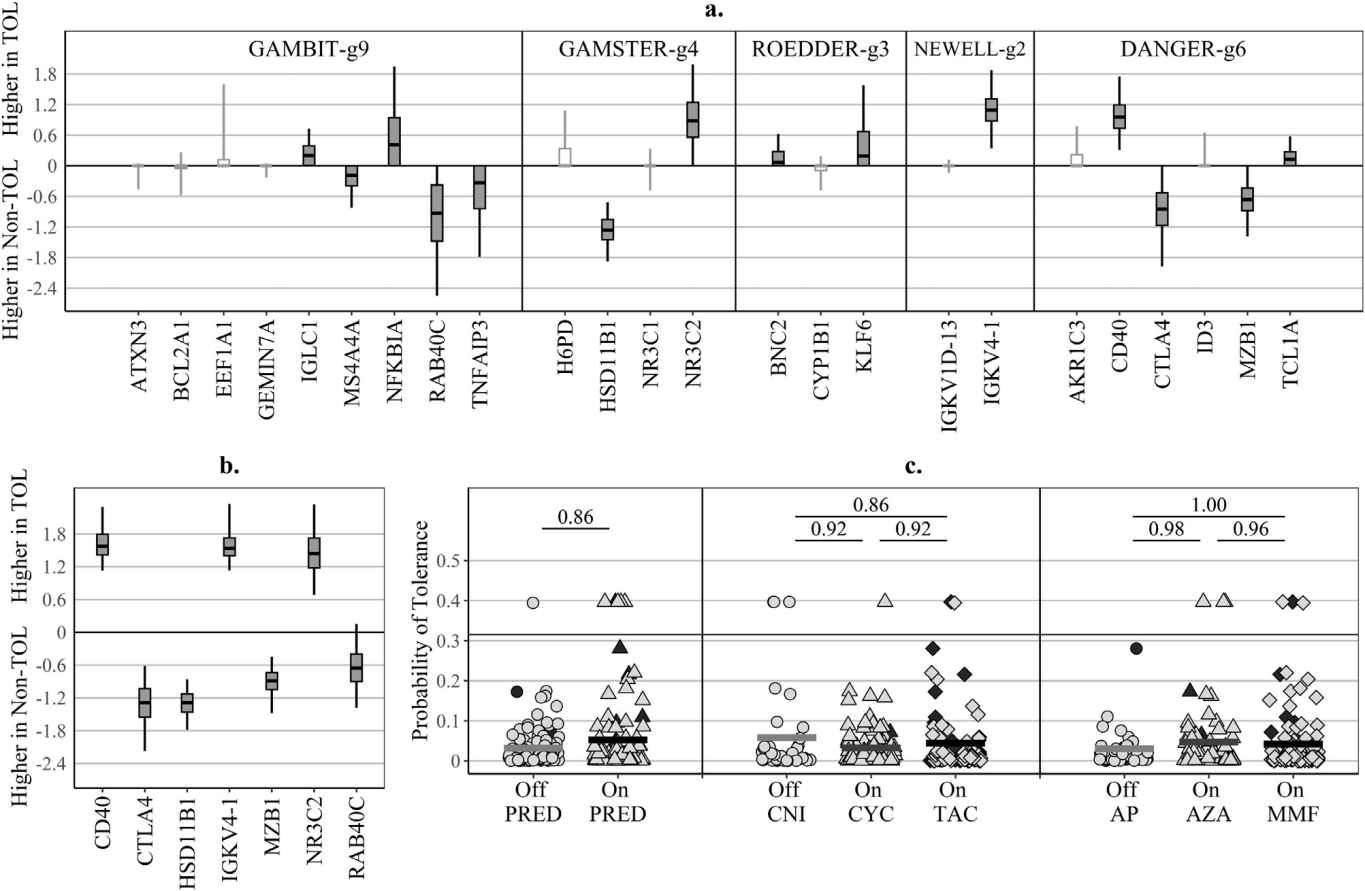
Combined signatures based on drug-adjusted gene-expression values. (**a**) **COMBINED-all** – regression coefficients for a signature including all genes examined in the study, with an elastic net penalty which favours gene exclusion (alpha=0⋅95); (**b**) **COMBINED-g7** – regression coefficients for a signature including the seven selected genes, with elastic net penalty which favours gene retention (alpha=0⋅05); (**c**) **COMBINED-g7** – influence of immunosuppressive drugs on the predicted probability of tolerance (percentage of explained variability R^2^≤1%); **Box and whiskers** – summary of regression coefficients of elastic net models derived from 100 repeats of six-fold cross-validation cycles (600 models in total) – horizontal line: median, box – 25^th^–75^th^ centile range; whiskers – 2⋅5^th^–97⋅5^th^ centile range; Genes with values closer to zero contributed less to the discrimination of operational tolerance; **White boxes** – genes with zero regression coefficients (i.e. excluded) from the final complete dataset model; **Grey boxes** – genes with non-zero regression coefficients (i.e. included) in the final complete dataset model; **Gene expression** – derived with the –ΔCt method, relative to hypoxanthine phosphoribosyl-transferase (HPRT) as a house-keeping gene (gene details are shown in Supplementary Table S1), with drug adjustment in linear models regressing gene-expression values for each gene on indicators of drug therapy: prednisolone (**PRED**) – off or on; calcineurin inhibitors (**CNI**) – off, on cyclosporine (**CYC**), or on tacrolimus (**TAC**); anti-proliferative agent (**AP**) – off, on azathioprine (**AZA**), or on mycophenolate mofetil (**MMF**).

Despite using a vigorous elastic net shrinkage penalty (alpha=0⋅95), 14 of all 24 genes included in the COMBINED-all signature showed a non-zero median of the elastic net regression coefficients obtained in cross-validation, with each of the five original signatures contributing genes and neither retaining all genes (Fig. 3a). Three genes: *HSD11B1, IGKV1–4,* and *CD40* stood out as the best candidates for a generalisable signature, as they were consistently retained in over 97⋅5% of the cross-validation elastic net models, followed by *RAB40C, NR3C2, CTL4,* and *MZB1,* which were retained in over 75% of the cross-validation models (Fig. 3a). OT discrimination was retained when the gene-set was confined to these seven genes, with some marginal improvement (Table 3). The probabilities of OT derived from the parsimonious COMBINED-g7 signature were not associated with IS drugs (Fig. 3c). There was good agreement in the identification of TOL-positivity between T1-cohort and T2-cohort (kappa=0⋅65 for COMBINED-all and COMBINED-g7) (Table 4). COMBINED-g7 identified as TOL-positive at both timepoints six of the 12 TOL patients and a single stable patient, receiving PRED, CYC, and MMF (Supplementary Fig. S6). This stable patient was also identified as TOL-positive by the drug-adjusted versions of GAMBIT-g9, NEWELL-g2, and DANGER-g6 (Supplementary Table S2).

Supplementary Table S3 contains the regression coefficients for all examined signatures. The legend of this table provides detailed instructions on how to use the regression coefficients to perform drug-adjustment of gene expression values and to calculate probabilities of OTSupplementary Material file.

## 4 Discussion

To our knowledge, this is the first direct comparison of gene-expression signatures of OT in KTRs in the laboratory, using a relatively large cohort of patients. We demonstrated that IS drugs considerably influence gene-expression and the probability of OT predicted with signatures based on gene-expression unadjusted for IS drugs. We developed the first parsimonious consensus signature based on drug-adjusted gene-expression values, which includes the strongest predictors among 24 genes from five previously published signatures.

The influence of IS drugs was particularly notable for ROEDDER-g3, for which the statistical selection of the signature gene-set, comprising genes related to monocyte-derived dendritic cells, did not account for the effect of IS drugs. Although the authors have considered the possibility of IS drug regimens influencing their signature, they could not find statistically significant differences between the drug regimens of 11 treated KTRs identified as TOL-positive [12]. Nevertheless, we have now demonstrated that the probability of OT based on ROEDDER-g3 is lower with AZA therapy and that OT discrimination was essentially lost when using drug-adjusted gene-expression values. Lee et al. have also failed to find differential expression of the three signature genes in Korean KTRs, in a dataset including eight TOL KTRs [21].

The development of DANGER-g6, a signature dominated by B-cell genes, has similarly relied on a statistical procedure using unadjusted gene-expression values [10]. Although the authors concluded that their signature is independent of IS drugs, we have now shown that PRED and AZA considerably influence the predicted probabilities of OT. There are several possible explanations for this discrepancy. First, we have adjusted the association of individual IS drugs for other drugs included in the IS regimen, while Danger et al. compared groups on/off individual drugs [10]. Considering all IS drugs is important, because patients off one IS drug would most likely be receiving another. GAMBIT patients off PRED were, indeed, treated preferentially with CNI, so a simple comparison on/off PRED, without adjustment for CNI therapy, would have been a comparison between PRED and CNI. Furthermore, Danger et al. [10] grouped AZA and MMF when comparing off/on AP agents, while we found that AZA was the main drug affecting the signature, so the proportion of AZA-treated KTRs would influence the joint effect in a combined AP group. In addition, Danger et al. used isolated PBMC from blood collected in vacutainers with ethylenediamine tetraacetic acid [10], while we collected whole blood in Tempus™ tubes in GAMBIT.

Another outstanding example illustrating the substantial influence of IS drugs on gene-expression signatures of OT developed using statistical algorithms for gene selection is the complete lack of overlap between the two signatures derived in our group from the same microarray data (the RISET study) using initially unadjusted gene-expression and later using drug-adjusted gene-expression values for GAMBIT-g9 [6,22]. The former signature was, indeed, influenced by IS regimens [6,8].

Although the AUC for NEWELL-g2, a signature including B-cell genes, was not influenced by drug-adjustment, the predicted probability of OT was considerably lower with PRED and MMF and higher with CNI therapy, in agreement with the findings of Bottomley et al. [8]. Moreso et al. had earlier reported higher peripheral expression of the *IGKV1D-13* and *IGKV4–1* genes in CNI treated KTRs, especially with longer treatment (5–10 years) [17]. Although the authors additionally reported lower expression levels of the two B-cell genes in AZA treated KTRs, their findings were based only on 8 stable KTRs and we could not confirm this observation with 67 stable KTRs. Asare et al. have similarly reported that TOL-positivity was higher with TAC therapy and was lower with PRED, MMF, thymoglobuline, and mTOR-inhibitor therapy [23]. The authors additionally reported increased counts for all B-cell sub-types in KTRs consistently TOL-positive compared to TOL-negative KTRs, but without the characteristic differential increase in transitional and naïve B-cells described in KTRs with OT [23]. This would support a hypothesis that the observed differences between TOL-positive and TOL-negative KTRs were partly determined by IS drugs rather than OT. We and others have previously shown that transitional B-cells are in fact suppressed by AZA and PRED, with a dose response effect for PRED and an increase in transitional B-cell counts after PRED withdrawal [6,8]. We have now demonstrated that 30% of the variability of the B-cell genes *IGKV1D-13* and *IGKV4–1* can be explained by drug therapy, which supports the concept that the effects of IS drugs on gene expression in peripheral blood and on signatures of OT reflect the effect of drugs on circulating immune cells. Furthermore, despite NEWELL-g2 predicting higher probability of OT for KTRs with TAC therapy and Moreso et al. concluding that CNI treated KTRs consistently express biomarkers associated with true tolerance [17], TAC withdrawal in clinical trials has dramatically failed [24,25]. Therefore, a gene-expression signature unadjusted for IS drug therapy does not necessarily identify a drug-independent B-cell pattern which would be maintained after IS reduction or withdrawal. Accordingly, we argue that drug adjustment may facilitate a distinction between underlying biological differences and the effects of IS drugs.

We have shown that five completely different gene-sets can all discriminate untreated TOL KTRs from treated Non-TOL KTRs. This, however, is not the distinction of interest. To be applicable in clinical practice, a gene-expression signature of OT should identify molecular features of OT among treated KTRs. Although Asare et al. have emphasised that NEWELL-g2 identifies KTRs without donor-specific antibodies (DSA) and with high and stable eGFR [23], a stable graft function, and absence of DSA in treated KTRs may be dependent on maintaining IS therapy [5]. A signature of OT would, therefore, need to discriminate between stability resulting from IS therapy, which would be lost, and stability resulting from OT, which could be maintained after IS withdrawal. To address this, one would ideally need to examine molecular features of treated stable KTRs at baseline and then follow them up after IS drug withdrawal to establish the outcome. However, while this experimental paradigm has been successfully implemented in liver transplantation [26], attempts of complete IS withdrawal in kidney transplantation have failed [24]. Therefore, *in silico* IS drug withdrawal with a statistical adjustment for IS drug therapy could be used as an approximation to experimental IS withdrawal in the signature development stage. Although most experts would accepts that IS drugs influence gene expression [7], the influence of IS drugs on signatures of OT is often examined only after selecting the signature gene-set [27]. We have now demonstrated that adjustment for IS drug therapy prior to gene selection can minimise the drug dependence of signatures of OT. We have also shown that lack of adjustment can result in the statistical selection of genes such as the *BNC2* gene, which are differentially expressed in TOL KTRs mainly due to IS drug therapy. Ultimately, a clinically applicable signature of OT would need to be validated with prospective IS drug reduction in a clinical trial. An *in silico* approach is not a substitute for a rigorous validation. Nevertheless, advancing a signature of OT to a clinical trial stage in the absence of any mechanism to account for the influence of IS drugs in the signature development stage, would be a considerable sacrifice to ask from patients.

Although sceptics may question the future of gene-expression signatures of OT in kidney transplantation, there is a pressing need for personalisation of IS regimens for precision medicine therapies. Gene-expression signatures of OT could gain more credibility, if developed with joint analysis of published signatures in a new dataset and selection of consensus gene-sets including the strongest predictors. This could reduce the multiplicity of signatures and improve their generalisability. Danger et al. have adopted the joint analysis approach with unadjusted gene-expression levels [10], selecting the six strongest predictors among 20 consensus genes of OT in kidney and liver transplantation, previously identified by Baron et al. [28]. We have now extended this approach to drug-adjusted gene expression. Our COMBINED-g7 signature is a parsimonious consensus gene-set which captures the strongest predictors of OT after minimising the influence of IS drugs. A parsimonious gene-set is cost-effective, as it includes the smallest number of genes, which retain the predictive performance of an extended gene-set. We have shown that very few stable patients are identified as tolerant, which would account for the numerous failures of grafts after IS withdrawal [24], and the scarcity of patients that have achieved OT. Although we have identified in this study the genes consistently affected in most examined KTRs, an external validation of our COMBINED-g7 signature in an independent dataset would be required prior to considering a further validation in a clinical trial.

A biological rationale behind the genes included in COMBINED-g7 is supported by laboratory studies. First, transitional B-cells have been associated with protection from acute rejection and their involvement in OT [29,30], as well as that of B-cell genes such as *IGKV4–1,* is well established [27]. Further, we and others have shown that KTRs with established OT, in contrast to CRs, display a fully functional CD40/STAT3 (signal transducer and activator of transcription 3) signalling pathway in regulatory B-cells [31,32]. An antibody blockage of *CD40* gene has, indeed, abrogated the development of tolerance in an experimental mixed-chimerism model of kidney transplant tolerance induction [33]. In addition, *CTLA4* gene plays a key role in the activation of regulatory T-cells (Tregs) involved in OT, but also of conventional T-cells. A positive association of *CTLA4* gene with acute rejection episodes in humans has been described [34], matching the higher levels of expression we observed in Non-TOL compared to TOL KTRs. A deletion of the equivalent mouse gene *Ctla4* during adulthood was associated with expansion of conventional, as well as Tregs, which retained their IS properties [35]. Furthermore, we have previously argued that the downregulation of the pro-inflammatory mineralocorticoid receptor gene *NR3C2* and the upregulation of the anti-inflammatory cortisol-activating enzyme gene *HSD11B1* in stable and CR KTRs is likely a response to the immunological challenge presented by the kidney allograft, which was not observed in KTRs with OT [9]. Moreover, the silencing of the *MZB1* gene with methylation described in hepatocellular and gastric cancers could be seen as a form of immunological tolerance [36,37], while MZB1 protein was increased in B-cells from patients with the autoimmune condition systemic lupus erythematosus [38]. Finally, RAB40C protein regulates, via an ubiquitin-proteasome system, the degradation of RACK1 protein, which is important in tumour growth and T-cell migration [39]. The *RAB40C* gene was also among the main genes silenced with methylation in breast cancer [40], matching the lower expression in TOL compared to Non-TOL KTRs that we observed. There are, therefore, sound reasons to believe that the seven consensus genes are biologically, as well as statistically important for OT.

Our study has several limitations which must be acknowledged. These, most importantly, include the lack of external validation in an independent dataset and the smaller number of TOL KTRs (*n* = 18), compared to the 46 considered by Danger et al. [10], but also the lack of KTRs treated with mTOR inhibitors, and the smaller number of follow-up samples in the T2-cohort compared to the T1-cohort. Although the T2-cohort addresses the stability of the signature, it comprises the same patients as the T1-cohort and is not a substitute for an independent dataset. It should further be noted that KTRs from the GAMBIT study contributed to the development of the GAMBIT-g9 signature and informed the selection of the genes for the new consensus signature COMBINED-g7, as well as the drug-adjustment models, which may account for a better performance than it would be observed in an independent dataset. Nevertheless, the GAMBIT study provides an external validation dataset for the genes included in the remaining four signatures. Notably, Danger et al. have used the same patients as Baron et al. [10,28], which would be contributing to a degree of optimism in the reported predictive performance of their model-development set, including in the cross-validation that they have used. A further limitation was the lack of data on protein expression and enzyme activity, which precluded conclusions on whether the observed gene expression differences between TOL and Non-TOL KTRs were translated in downstream effects. Such mechanistic investigations, however, were beyond the scope of the current study. Furthermore, we did not have data to examine dose response associations with serum levels of CNI drugs. These, however, may not be completely reliable as they would depend on the time interval between drug intake and sample collection and the compliance of the patients with the recommended protocol. As the majority of KTRs in our study were white, a follow-up study in a dataset with larger ethnic diversity would be required for further validation of the generalisability of our newly identified signature. The strength of our study lies in the relatively large number of KTRs treated with a variety IS drug regimens, which is comparable to the 266 stable patients used by Danger et al. [10].

In conclusion, accounting for IS drug therapy prior to developing a gene-expression signature of OT is paramount and should be implemented before any signature is brought to evaluation in a biomarker-led clinical trial. Apparent molecular features of OT may otherwise be determined by treatment.

## Funding

The authors acknowledge financial support from FP7-HEALTH-2012-INNOVATION-1 (project number 305147: BIO-DrIM); 10.13039/501100000265Medical Research Council MRC grants to Maria P. Hernandez-Fuentes [G0801537/ID: 88245] and to MRC Centre for Transplantation [MRC grant no. MR/J006742/1]); Guy's and St Thomas’ Charity [grants R080530 and R090782]; NIHR-BRC School for Translational and Experimental Medicine/Cluster 4 Early Career Award in Translational Science to Sofia Christakoudi. Sofia Christakoudi, Irene Rebollo-Mesa, Paula Mobillo, and Daniel Stahl were also funded by the EU project BIO-DrIM. Estefania Nova-Lamperti was funded by a scholarship from CONICYT Bicentennial Becas-Chile, Chile. Maria P. Hernandez-Fuentes has also received funding from the 10.13039/501100004963European Union, Seventh Framework Programme [FP7/2007–2013], under grant agreement [No HEALTH-F5–2010–260687: The ONE Study]. Ondrej Viklický received funding from the Czech Ministry of Health [grant number NV19–06–00031]. The research was funded/supported by the National Institute for Health Research (NIHR) Biomedical Research Centre based at Guy's and St Thomas' NHS Foundation Trust and King's College London. The views expressed are those of the author(s) and not necessarily those of the NHS, the NIHR, or the Department of Health. All UK based centres received service support through Clinical Research Networks [study portfolio number 7521], this allowed the support of a large number of research nurses in the different centres, whose dedication has allowed the samples and clinical information to be collected. The study sponsors had no involvement in the study design; in the collection, analysis, and interpretation of the data; in the writing of the report; or in the decision to submit the paper for publication.

## Contributions

Sofia Christakoudi contributed to study conception and design, the statistical analysis, interpretation and drafting of the article; Manohursingh Runglall was instrumental for organising and performing the laboratory analysis and also contributed to study conception and design, interpretation of results, and drafting of the article; Irene Rebollo-Mesa contributed to study conception and design; Paula Mobillo, Tjir-Li Tsui, Estefania Nova-Lamperti, Catharine Taube, Sonia Norris, and Yogesh Kamra contributed to the laboratory analysis; Rachel Hilton, Titus Augustine, Sunil Bhandari, Richard Baker, David Berglund, Sue Carr, David Game, Sian Griffin, Philip A. Kalra, Robert Lewis, Patrick B. Mark, Stephen D. Marks, Iain MacPhee, William McKane, Markus G. Mohaupt, Estela Paz-Artal, Sui Phin Kon, Daniel Serón, Manish D. Sinha, Beatriz Tucker, and Ondrej Viklický contributed to patient recruitment and data collection; Daniel Stahl contributed to the statistical analysis strategy; Robert I. Lechler contributed to study conception and design; Graham M. Lord contributed to the interpretation of results; Maria P. Hernandez-Fuentes contributed to study conception and design, interpretation of results, and drafting of the article. All authors revised critically the manuscript for important intellectual content and approved the final submitted version. The corresponding author (Sofia Christakoudi) confirms that she had full access to all data in the study and had final responsibility for the decision to submit the manuscript for publication.

## Declaration of Competing Interest

Dr. Christakoudi reports grants from FP7-HEALTH-2012-INNOVATION-1 (project number 305147: BIO-DrIM), grants from National Institute for Health Research (NIHR) Biomedical Research Centre based at Guy's and St Thomas' NHS Foundation Trust and King's College London, during the conduct of the study. Mr. Runglall has nothing to disclose. Dr. Mobillo reports grants from FP7-HEALTH-2012-INNOVATION-1 (project number 305147: BIO-DrIM), during the conduct of the study. Dr. Rebollo-Mesa reports grants from [MR/J006742/1] to MRC Centre for Transplantation, grants from FP7-HEALTH-2012-INNOVATION-1 (project number 305147: BIO-DrIM), during the conduct of the study; other from UCB Pharma SRL, outside the submitted work. Mr. Tsui has nothing to disclose. Dr. Nova-Lamperti reports grants from CONICYT Bicentennial-Becas-Chile scholarship, during the conduct of the study. Dr. Taube has nothing to disclose. Dr. Norris has nothing to disclose. Mr. Kamra has nothing to disclose. Dr. Hilton reports personal fees from Chiesi Ltd, outside the submitted work. Dr. Augustine has nothing to disclose. Dr. Bhandari has nothing to disclose. Dr. Baker has nothing to disclose. Dr. Berglund has nothing to disclose. Dr. Carr has nothing to disclose. Dr. Game reports personal fees from Advisory board Chiesi pharmaceuticals, personal fees from Advisory board Recordati Rare Diseases, personal fees from Advisory board Syneos Health, outside the submitted work. Dr. Griffin has nothing to disclose. Dr. Kalra has nothing to disclose. Dr. Lewis has nothing to disclose. Dr. Mark reports personal fees and non-financial support from Vifor, personal fees from Astrazeneca, grants from Boehringer Ingelheim, personal fees and non-financial support from Pharmacosmos, personal fees from Janssen, personal fees from Novartis, personal fees from Pfizer, personal fees from Bristol Myers Squibb, personal fees and non-financial support from Napp, outside the submitted work. Dr. Marks has nothing to disclose. Dr. MacPhee reports other from AstraZeneca, other from AstraZeneca, grants and personal fees from Chiesi, personal fees from Astellas, personal fees from Sandoz, outside the submitted work. Dr. McKane has nothing to disclose. Dr. Mohaupt has nothing to disclose. Dr. Paz-Artal has nothing to disclose. Dr. Kon has nothing to disclose. Dr. Seron has nothing to disclose. Dr. Sinha has nothing to disclose. Dr. Tucker has nothing to disclose. Prof. Viklicky reports grants from CZECH MINISTRY OF HEALTH, during the conduct of the study. Dr. Stahl reports grants from FP7-HEALTH-2012-INNOVATION-1 (project number 305147: BIO-DrIM), during the conduct of the study. Dr. Lechler has nothing to disclose. Dr. Lord has nothing to disclose. Dr. Hernandez-Fuentes reports grants from FP7-HEALTH-2012-INNOVATION-1 (project number 305147: BIO-DrIM), grants from Medical Research Council MRC grants to Maria P. Hernandez-Fuentes [G0801537/ID: 88245], grants from Guy's and St Thomas’ Charity [grants R080530 and R090782], grants from EU; FP7/2007–2013], under grant agreement [No HEALTH-F5–2010–260687: The ONE Study], grants and non-financial support from National Institute for Health Research (NIHR) Biomedical Research Centre based at Guy's and St Thomas' NHS Foundation Trust and King's College London, non-financial support from Clinical Research Networks [study portfolio number 7521], during the conduct of the study; other from UCB Celltech., outside the submitted work; .

## Appendix Supplementary materials

Image, application 1

## Footnotes

Supplementary material associated with this article can be found, in the online version, at doi:10.1016/j.ebiom.2020.102899.
