# Supplementary material for: Development and validation of the first consensus gene-expression signature of operational tolerance in kidney transplantation, incorporating adjustment for immunosuppressive drug therapy
Source: eBioMedicine. 2020 Jul 21;58:102899. doi: 10.1016/j.ebiom.2020.102899 (PMC7374249; doi:10.1016/j.ebiom.2020.102899)
Supplement: Supplementary file 1 [file mmc1.pdf]

Supplementary Material

Development and validation of the first consensus gene-expression signature of operational tolerance in kidney transplantation, incorporating adjustment for immunosuppressive drug therapy

Supplementary Methods 3

Cross-validation algorithm 3

Supplementary Tables 5

Supplementary Table S1 Description of analysed genes 5

Supplementary Table S2 Kidney transplant recipients identified as TOL-positive at both time points 6

Supplementary Table S3 Regression coefficients for all examined signatures 7

Supplementary Figures 9

Supplementary Fig. S1a,b Influence of IS drugs on the individual gene-expression levels and the discrimination of operational tolerance *(continues on next page)* 9

Supplementary Fig. S1c,d Influence of IS drugs on the individual gene-expression levels and the discrimination of operational tolerance *(continues on next page)* 10

Supplementary Fig. S1e,f Influence of IS drugs on the individual gene-expression levels and the discrimination of operational tolerance *(continues on next page)* 11

Supplementary Fig. S1g,h Influence of IS drugs on the individual gene-expression levels and the discrimination of operational tolerance *(continues on next page)* 12

Supplementary Fig. S1i,j Influence of IS drugs on the individual gene-expression levels and the discrimination of operational tolerance *(continues on next page)* 13

Supplementary Fig. S1k,l Influence of IS drugs on the individual gene-expression levels and the discrimination of operational tolerance *(continues on next page)* 14

Supplementary Fig. S1m,n Influence of IS drugs on the individual gene-expression levels and the discrimination of operational tolerance *(continues on next page)* 15

Supplementary Fig. S1o,p Influence of IS drugs on the individual gene-expression levels and the discrimination of operational tolerance *(continues on next page)* 16

Supplementary Fig. S1q,r Influence of IS drugs on the individual gene-expression levels and the discrimination of operational tolerance *(continues on next page)* 17

Supplementary Fig. S1s,t Influence of IS drugs on the individual gene-expression levels and the discrimination of operational tolerance *(continues on next page)* 18

Supplementary Fig. S1u,v Influence of IS drugs on the individual gene-expression levels and the discrimination of operational tolerance *(continues on next page)* 19

Supplementary Fig. S1w,x Influence of IS drugs on the individual gene-expression levels and the discrimination of operational tolerance *(legend on next page)* 20

Supplementary Fig. S1 Influence of IS drugs on the individual gene-expression levels and the discrimination of operational tolerance *(legend)* 21

Supplementary Fig. S2a-f Influence of the dose of immunosuppressive drugs on unadjusted gene-expression levels *(continues on next page)* 22

Supplementary Fig. S2g-l Influence of the dose of immunosuppressive drugs on unadjusted gene-expression levels *(continues on next page)* 23

Supplementary Fig. S2m-r Influence of the dose of immunosuppressive drugs on unadjusted gene-expression levels *(continues on next page)* 24

Supplementary Fig. S2s-x Influence of the dose of immunosuppressive drugs on unadjusted gene-expression levels *(continues on next page)* 25

Supplementary Fig. S2y-ad Influence of the dose of immunosuppressive drugs on unadjusted gene-expression levels *(legend)* 26

Supplementary Fig. S3a-f Group discrimination achieved by the unadjusted and the drug-adjusted calibration version of gene-expression signatures *(continues on next page)* 28

Supplementary Fig. S3g-l Group discrimination achieved by the unadjusted and the drug-adjusted calibration version of gene-expression signatures 29

Supplementary Fig. S4 Comparison of estimated glomerular filtration rate (eGFR) between TOL-positive or TOL-negative patients 30

Supplementary Fig. S5. Influence of the dose of immunosuppressive drugs on the predicted probability of tolerance. 31

Supplementary Fig. S6 Predicted probability of operational tolerance (OT) for patients with samples collected at both time points 1 and 2 32

Supplementary Methods

Cross-validation algorithm

Cross-validation included the following steps:

1. The complete T1-cohort dataset of kidney transplant recipients (KTRs) was split in six parts at random (in strata by patient type). Each part included 3 of the tolerant (TOL) (18/6=3), 31 of the stable (ST) (186/6=31) and 6 of the chronic rejectors (CR), except for the sixth part, which included only 4 CR (34/6=5·67, which rounds to 6 individuals).
2. One of the six parts was left out as a test subset and the remaining five parts constituted the training subset.
3. Non-TOL KTRs, i.e. ST + CR, from the training subset were separated and outliers of gene-expression levels were recoded to the next highest or lowest value, in order to avoid leverage of outliers on the regression coefficients in Step 4. Outliers were considered gene-expression levels with p-value<0.05 obtained from outliers test (*outlierTest* function, ***car*** package in R) with Bonferroni adjustment for the number of individuals in the group.
4. **Drug-adjustment linear regression models** were built for the Non-TOL KTRs of the training subset, including gene-expression for each individual gene as an outcome and categorical indicators of drug therapy as predictors (PRED=0 off prednisolone, PRED=1 on prednisolone; CNI=0 off calcineurin inhibitors, CNI=1 on cyclosporine, CNI=2 on tacrolimus; AP=0 off anti-proliferative drugs, AP=1 on azathioprine, AP=2 on mycophenolate-mofetil, where 0, 1 and 2 are categories and not numbers).

$$\mathbf{-\Delta Ct_{\mathit{GENE}} \sim PRED + CNI + AP}$$

   The $\mathbf{R^2}$ statistics derived from these models represented the percentage of gene-expression variability explained by drug therapy.
5. **Drug-adjusted gene-expression levels** were calculated for all KTRs (training and test subsets, TOL and Non-TOL) as the residuals of the models from Step 4, i.e. as the difference between the value observed for $-\Delta Ct_{GENE}$ and the value predicted from the model in Step 4. For TOL KTRs: PRED=0, CNI=0 and AP=0.
6. **Missing values** were imputed ("impute" package) in the complete training subset (TOL and Non-TOL). Imputation for the test subset was performed by adding each test sample individually to the completed training matrix, in order to ensure that the imputation rules would be the same for any test sample, i.e. would not be influences by other test samples.
7. **Outliers** of drug-adjusted gene-expression levels for the complete training subset (TOL and Non-TOL) were recoded to the next highest or lowest value, in order to avoid leverage of outliers on the elastic net regression coefficients in Step 9.
8. **The penalty parameter** lambda for elastic net regression was optimised as the median of 100 repeats of six-fold cross-validation cycles within the training subset defined in Step 2. Note that this is a separate cross-validation in which the training subset is further divided to training and test subsets. This procedure is incorporated in function "cv.glmnet" of package "glmnet".
9. **Elastic net regression** was performed within the training subset ("glmnet" package), using the penalty parameter lambda derived in Step 8 and setting the penalty parameter alpha to 0·05 (similar to ridge regression), which was not aimed at gene selection and largely preserved all genes, but allowed exclusion only of completely uninformative genes, by shrinking their regression coefficients to zero.
10. **Predicted probabilities of operational tolerance** (OT) were generated for the samples in the test subset, based on the elastic net models defined in Step 9.
11. Steps 2 to 10 were repeated, each time leaving out a new one-sixth part as a test subset and using the remaining five-sixths as a training subset, until each part and each sample had been used once and only once as a test subset. This generated one set of predicted probabilities for the complete T1-cohort. In this set, the predicted probability of OT for each individual patient was derived from a model in the training of which this patient had not participated.
12. **Cross-validated AUC** (area under the receiver operating characteristics curve, with 95% DeLong confidence interval) and **cross-validated specificity** were calculated for the complete set of predicted probabilities of OT from Step 11, using as a cut-off the median of the predicted probabilities of OT for all TOL KTRs in T1-cohort ("pROC" package).
13. Steps 1 to 12 were repeated 100 times, with a new random seed for each split in Step 1, thus generating 100 sets of predicted probabilities of OT for T1-cohort (100 values for each patient in T1-cohort), 600 drug-adjustment linear regression models, with 600 corresponding sets of regression coefficients and 600

corresponding values for the R^2 statistic for each gene in the signature, and 600 elastic net models with the corresponding regression coefficients.

14. Repeats from Step 13 were summarised with median and 2·5th–97·5th centile range and, for summaries of elastic net regression coefficients, also 25th–75th centile range. The medians derived for the regression coefficients in this step, included in Supplementary Table S3, were used in the final model for each signature.
15. Steps 1 to 14 were repeated for each signature, using the genes specified in Table 1.
16. For T2-cohort and healthy controls probabilities of OT were calculated using the final model for each signature, derived in Step 14.

Note 1: For the unadjusted version of each signature, Steps 3 to 5 were omitted.

Note 2: For gene selection in the COMBINED-all signature, Steps 1 to 14 were repeated using drug-adjusted gene expression for all genes examined in this study and setting the penalty parameter alpha in Step 9 to 0·95 (close to lasso regression), which enforces vigorous shrinkage of the elastic net regression coefficients.

Supplementary Tables

Supplementary Table S1 Description of analysed genes

	Gene symbol	Gene Name	Assay ID	Gene ID
GAMBIT-g9				
Genes	*ATXN3*	Ataxin 3	Hs01026447_m1	4287
	BCL2A1	BCL2-related protein A1	Hs00187845_m1	597
	EEF1A1	Eukaryotic translation elongation factor 1 alpha 1	Hs00265885_g1	1915
	GEMIN7	Gem (nuclear organelle) associated protein 7	Hs00226769_m1	79760
	IGLC1	Immunoglobulin lambda constant 1	Hs00760769_s1	3537
	MS4A4A	Membrane-spanning 4-domains, subfamily A, member 4A	Hs01106863_m1	51338
	NFKBIA	Nuclear factor of kappa light polypeptide gene enhancer in B-cells inhibitor, alpha	Hs00153283_m1	4792
	RAB40C	RAB40C, member RAS oncogene family	Hs00368350_m1	57799
	TNFAIP3	Tumor necrosis factor, alpha-induced protein 3alpha	Hs00234713_m1	7128
Reference	*HPRT*	Hypoxanthine phosphoribosyltransferase 1	Custom assay	3251
GAMSTER-g4				
Genes	*H6PDH*	Hexose-6-phosphate dehydrogenase (glucose 1-dehydrogenase)	Hs00188728_m1	9563
	HSD11B1	Hydroxysteroid (11-beta) dehydrogenase 1	Hs01547870_m1	3290
	NR3C1	Nuclear receptor subfamily 3, group C, member 1 (glucocorticoid receptor, GR)	Hs00353740_m1 (GRα+β isoforms)	2908
	NR3C2	Nuclear receptor subfamily 3, group C, member 2 (mineralocorticoid receptor, MR)	Hs01031809_m1	4306
Reference	*HPRT*	Hypoxanthine phosphoribosyltransferase 1	Custom assay	3251
ROEDDER-g3				
Genes	*BNC2*	Basonuclin 2	Hs00417700_m1	54796
	CYP1B1	Cytochrome P450 family 1 subfamily B member 1	Hs00164383_m1	1545
	KLF6	Kruppel like factor 6	Hs00810569_m1	1316
Reference	*HPRT*	Hypoxanthine phosphoribosyltransferase 1	Custom assay	3251
NEWELL-g2				
Genes	*IGKV1D-13*	Immunoglobulin kappa variable 1D-13	Hs04272697_g1	28902
	IGKV4-1	Immunoglobulin kappa variable 4-1	Hs04272696_g1	28908
Reference	*GAPDH*	Glyceraldehyde-3-phosphate dehydrogenase	Hs99999905_m1	2597
DANGER-g6				
Genes	*AKR1C3*	Aldo-keto reductase family 1, member C3	Hs00366267_m1	8644
	CD40	CD40 molecule, TNF receptor superfamily member 5	Hs00374176_m1	958
	CTLA4	Cytotoxic T-lymphocyte associated protein 4	Hs00175480_m1	1493
	ID3	Inhibitor of DNA binding 3, HLH protein	Hs00171409_m1	3399
	MZB1	Marginal zone B and B1 cell specific protein	Hs00414907_m1	51237
	TCL1A	T-cell leukemia/lymphoma 1A	Hs00172040_m1	8115
Reference	*ACTB*	Actin beta	Hs99999903_m1	60
	B2M	Beta-2-microglobulin (Beta2M)	Hs00984230_m1	567
	GAPDH	Glyceraldehyde-3-phosphate dehydrogenase	Hs99999905_m1	2597
	HPRT1	Hypoxanthine phosphoribosyltransferase 1	Hs99999909_m1	3251
COMBINED-g7				
Genes	*CD40*	CD40 molecule, TNF receptor superfamily member 5	Hs00374176_m1	958
	CTLA4	Cytotoxic T-lymphocyte associated protein 4	Hs00175480_m1	1493
	HSD11B1	Hydroxysteroid (11-beta) dehydrogenase 1	Hs01547870_m1	3290
	IGKV4-1	Immunoglobulin kappa variable 4-1	Hs04272696_g1	28908
	MZB1	Marginal zone B and B1 cell specific protein	Hs00414907_m1	51237
	NR3C2	Nuclear receptor subfamily 3, group C, member 2 (mineralocorticoid receptor, MR)	Hs01031809_m1	4306
	RAB40C	RAB40C, member RAS oncogene family	Hs00368350_m1	57799
Reference	*HPRT*	Hypoxanthine phosphoribosyltransferase 1	Custom assay	3251

Gene symbol – gene abbreviation; **Gene name** – full gene name (alternative abbreviation/name); **Assay ID** – assay number in Applied Biosystems (Thermo Fisher Scientific, UK); **Reference** – used as house-keeping gene, as described in Table 1; **Gene ID** – Gene number in NCBI (National Center for Biotechnology Information, U.S. National Library of Medicine, https://www.ncbi.nlm.nih.gov/gene/); **Custom assay -** sequences have previously been reported in reference (6); **Signatures** – publications (reference in main manuscript): GAMBIT-g9 (6); GAMSTER-g4 (9); ROEDDER-g3 (12); NEWELL-g2 (11); DANGER-g6 (10).

Supplementary Table S2 Kidney transplant recipients identified as TOL-positive

PATIENT	GAMBIT	GAMSTER	ROEDDER	NEWELL	NEWELL	DANGER	DANGER	COMBINED	Immunosuppressive		
	g9	g4	g3	g2	g2	g6	g6	g7		drugs	
Drug adj.	yes	yes	no	no	yes	no	yes	yes	PRED	CNI	AP
TOL1	+/+	+/+	-	+/+	+/+	+	+/+	+/+	-	-	-
TOL2	+/+	-	-	+/+	+/+	+/+	+/+	+	-	-	-
TOL3	+/+	-	+	+	+	+	-	+/+	-	-	-
TOL4	+/+	-	-	+	+	+	+/+	+/+	-	-	-
TOL5	+	+/+	+/+	+	+	+/+	+/+	+/+	-	-	-
TOL6	-	+/+	+/+	+	+	-	-	+	-	-	-
TOL7	-	+/+	+	+/+	+/+	-	-	+/+	-	-	-
TOL8	+	+/+	+	+/+	+/+	+	+/+	+/+	-	-	-
TOL9	+	-	+/+	-	+	+	+/+	+	-	-	-
TOL10	-	-	+/+	-	-	+	-	-	-	-	-
TOL11	+	-	-	-	+	+/+	+/+	+	-	-	-
ST1	+/+	+/+	-	-	-	-	-	-	PRED	-	MMF
ST2	+/+	+	-	-	+/+	-	+/+	+/+	PRED	CYC	MMF
ST3	+/+	-	-	-	-	-	-	-	-	CYC	MMF
ST4	-	+/+	-	-	-	-	-	-	PRED	CYC	MMF
ST5	-	+/+	-	-	-	-	-	-	PRED	-	AZA
ST6	-	+/+	-	-	-	-	-	-	-	CYC	AZA
ST7	-	-	+/+	+/+	+	-	-	-	-	CYC	AZA
ST8	-	-	+/+	+	+	-	-	-	-	TAC	-
ST9	-	-	+/+	-	-	-	-	-	PRED	CYC	-
ST10	-	-	+/+	+	+	-	-	-	PRED	TAC	-
ST11	-	+	-	+/+	-	-	-	-	-	TAC	AZA
ST12	-	-	-	+	+/+	-	-	-	-	TAC	MMF
CR1	+	-	-	+/+	+/+	-	+	-	PRED	TAC	AZA
CR2	+	+	-	+	+/+	-	-	-	PRED	TAC	-
CR3	-	-	-	+	-	-	+/+	-	-	TAC	AZA

Patient type: **TOL** – kidney transplant recipients (KTRs) with operational tolerance (OT); **ST** – stable KTRs; **CR** – chronic rejector KTRs; **TOL-positivity**: "+/+" – KTRs identified as TOL-positive at both time point 1 (T1-cohort) and time point 2 (T2-cohort), using as a cut-off the median predicted probability of OT in all TOL KTRs from the complete T1-cohort; **"+"** – KTRs identified as TOL-positive at only one of the time points; **"-"** – KTRs identified as TOL-negative at both time points; **Immunosuppressive (IS) drugs**: **AP** – anti-proliferative agents; **AZA** – azathioprine; **CNI** – calcineurin inhibitors; **CYC** – cyclosporine; **MMF** – mycophenolate mofetil; **PRED** – prednisolone; **TAC** – tacrolimus. Signature gene-sets and reference genes are described for each signature in Table 1. Gene-expression, as originally intended, was unadjusted for ROEDDER-g3, NEWELL-g2 and DANGER-g6 and drug-adjusted for GAMBIT-g9, GAMSTER-g4 and COMBINED-g7. The drug-adjusted versions of NEWELL-g2 and DANGER-g6 are included for comparison. The drug-adjusted versions of ROEDDER-g3 is omitted because it failed to achieve OT discrimination. COMBNED-all and the unadjusted versions of GAMBIT-g9 and GAMBIT-g4 are omitted because they are not recommended for practical application.

Supplementary Table S3 Regression coefficients for all examined signatures

SIGNATURE	DRUG ADJUSTMENT COEFFICIENTS						ELASTIC NET COEFFICIENTS		
**GAMBIT-g9**								**Drug-adjusted**	**Unadjusted**
	**A_DRUGS**	**B_PRED**	**B_CYC**	**B_TAC**	**B_AZA**	**B_MMF**	**A_ELNET**	-3.4754895	-5.1542284
**ATXN3**	1.5385908	0.0784094	-0.1579399	-0.0128879	0.0444260	0.1704071	**B_ATXN3**	0.5804705	-0.1608784
**BCL2A1**	4.1452890	0.3553253	-0.2625189	-0.3114506	0.0601610	0.2505673	**B_BCL2A1**	-0.4619760	-0.1605465
**EEF1A1**	9.6684721	-0.1507047	-0.0192796	0.0494189	0.0652415	0.0314436	**B_EEF1A1**	0.4304628	0.2699386
**GEMIN7A**	-0.4003678	0.1015581	-0.0930228	0.0031461	-0.0333415	0.0668394	**B_GEMIN7A**	-0.0032050	-0.5066101
**IGLC1**	-3.2985743	-1.4618883	0.6386798	1.1258282	-0.9704703	-0.5859626	**B_IGLC1**	0.7528207	0.7340473
**MS4A4A**	-1.2666096	-0.0450387	-0.1400766	-0.3015708	0.0740446	0.1083538	**B_MS4A4A**	-0.3229881	-0.0808614
**NFKBIA**	5.6091792	0.5518076	-0.2175926	-0.0518057	-0.0957182	0.2219800	**B_NFKBIA**	2.1500631	0.5118497
**RAB40C**	1.0371634	0.1058014	-0.2067216	-0.0478142	-0.0969775	-0.0191308	**B_RAB40C**	-2.2673832	-0.5877397
**TNFAIP3**	4.0287770	0.2418018	-0.2016792	-0.1199907	-0.1313204	0.0742238	**B_TNFAIP3**	-0.8398390	0.0271441
**GAMSTER-g4**								**Drug-adjusted**	**Unadjusted**
	**A_DRUGS**	**B_PRED**	**B_CYC**	**B_TAC**	**B_AZA**	**B_MMF**	**A_ELNET**	-2.9797996	-2.2918889
**H6PD**	2.8049911	0.2261150	-0.0498060	-0.0301939	-0.0704293	0.0155464	**B_H6PD**	0.2813327	0
**HSD11B1**	-5.1892391	-0.4698871	-0.1282591	-0.0412242	-0.0848157	-0.0335317	**B_HSD11B1**	-1.0394460	-0.3924504
**NR3C1**	3.6373785	0.1270446	-0.1273655	-0.0700048	0.0192701	0.1256151	**B_NR3C1**	-0.6645408	-0.6426652
**NR3C2**	-0.9253023	-0.3058538	0.2362751	0.2609131	0.1533608	0.0454577	**B_NR3C2**	1.0973116	0.2710949
**ROEDDER-g3**								**Drug-adjusted**	**Unadjusted**
	**A_DRUGS**	**B_PRED**	**B_CYC**	**B_TAC**	**B_AZA**	**B_MMF**	**A_ELNET**	-2.5123056	3.9318274
**BNC2**	-1.8805567	-0.1137042	-0.4360514	-0.1251796	-1.5389873	-0.5028989	**B_BNC2**	0	0.9565287
**CYP1B1**	2.2016147	0.1165295	-0.2700292	-0.1442503	0.2071730	0.1547046	**B_CYP1B1**	-0.0067018	-0.3923639
**KLF6**	5.9876412	0.2789714	-0.0725928	0.0309186	0.0226268	0.1593628	**B_KLF6**	0	-0.5481233
**NEWELL-g2**								**Drug-adjusted**	**Unadjusted**
	**A_DRUGS**	**B_PRED**	**B_CYC**	**B_TAC**	**B_AZA**	**B_MMF**	**A_ELNET**	-2.9470951	-0.7038864
**IGKV1D-13**	-3.2411228	-1.0121868	0.8558861	1.0227814	-0.1325289	-0.9907992	**B_IGKV1D-13**	-0.5277266	0.0749690
**IGKV4-1**	-4.6988220	-0.9720911	1.0676928	1.2243005	-0.0355029	-1.0861046	**B_IGKV4-1**	1.1542454	0.3600116
**DANGER-g6**								**Drug-adjusted**	**Unadjusted**
	**A_DRUGS**	**B_PRED**	**B_CYC**	**B_TAC**	**B_AZA**	**B_MMF**	**A_ELNET**	-3.0566766	17.8852049
**AKR1C3**	-9.1455784	-0.3026504	-0.2342605	-0.2084997	-0.8553879	-0.2922334	**B_AKR1C3**	0	0.8944277
**CD40**	-6.6125325	-0.3604262	0.1663179	0.3897970	-0.3928819	0.0346532	**B_CD40**	0.9585938	0.8493481
**CTLA4**	-7.3397077	-0.4071331	-0.2169585	-0.2585642	-0.0463473	-0.2076935	**B_CTLA4**	-0.8059301	0.7347183
**ID3**	-6.0399278	-0.7569557	0.4317041	0.6697880	-0.0068837	-0.0510149	**B_ID3**	0.2638415	-0.7256921
**MZB1**	-7.8053460	-0.9088747	0.5077987	0.5298233	0.1895265	-1.0837066	**B_MZB1**	0.1115602	0.3012439
**TCL1A**	-5.6665819	-1.3408617	0.2865060	0.7546753	-1.9413182	0.2607255	**B_TCL1A**	0.3603260	0.4687336
**COMBINED-all ***								**Drug-adjusted**	
	**A_DRUGS**	**B_PRED**	**B_CYC**	**B_TAC**	**B_AZA**	**B_MMF**	**A_ELNET**	-4.3135687	
**IGLC1**	-3.2922979	-1.4670951	0.6348009	1.1194817	-0.9715865	-0.5855817	**B_IGLC1**	0.2002007	
**MS4A4A**	-1.2651616	-0.0458689	-0.1397976	-0.2972779	0.0762380	0.1067079	**B_MS4A4A**	-0.1902464	
**NFKBIA**	5.6070183	0.5506004	-0.2207886	-0.0523206	-0.0953138	0.2221766	**B_NFKBIA**	0.4090685	
**RAB40C**	1.0398300	0.1053764	-0.2083370	-0.0488843	-0.0932342	-0.0145531	**B_RAB40C**	-0.9258964	
**TNFAIP3**	4.0282683	0.2396946	-0.2045102	-0.1259136	-0.1290232	0.0738044	**B_TNFAIP3**	-0.3345177	
**HSD11B1**	-5.1880036	-0.4696948	-0.1305009	-0.0506154	-0.0846368	-0.0345380	**B_HSD11B1**	-1.2575762	
**NR3C2**	-0.9247928	-0.3053883	0.2390384	0.2606242	0.1534882	0.0458926	**B_NR3C2**	0.8826016	
**BNC2**	-1.8822076	-0.1070188	-0.4355772	-0.1200669	-1.5432152	-0.5008193	**B_BNC2**	0.0635734	
**KLF6**	5.9865982	0.2750560	-0.0727871	0.0305807	0.0233118	0.1625895	**B_KLF6**	0.1936261	
**IGKV4-1**	2.3282610	-0.7805116	0.7563513	1.0444205	0.1562323	-0.9503348	**B_IGKV4-1**	1.0940359	
**CD40**	0.6485746	-0.2762314	-0.0261258	0.3188318	-0.3117541	0.1207561	**B_CD40**	0.9560463	
**CTLA4**	-0.0630898	-0.3353475	-0.4137458	-0.3397481	0.0319844	-0.1341183	**B_CTLA4**	-0.8493930	
**MZB1**	-0.5283354	-0.8197208	0.2700663	0.4203213	0.3026625	-0.9906421	**B_MZB1**	-0.6621875	
**TCL1A**	1.6395183	-1.2478498	0.0483737	0.6362777	-1.8386817	0.3540876	**B_TCL1A**	0.1266426	
**COMBINED-g7**								**Drug-adjusted**	
	**A_DRUGS**	**B_PRED**	**B_CYC**	**B_TAC**	**B_AZA**	**B_MMF**	**A_ELNET**	-4.3808099	
**CD40**	0.6477830	-0.2759510	-0.0274228	0.3205161	-0.3139046	0.1224405	**B_CD40**	1.5764386	
**CTLA4**	-0.0607098	-0.3346937	-0.4147388	-0.3414341	0.0319844	-0.1352440	**B_CTLA4**	-1.2889128	
**HSD11B1**	-5.1892391	-0.4698871	-0.1282591	-0.0412242	-0.0848157	-0.0335317	**B_HSD11B1**	-1.2889968	
**IGKV4-1**	2.3384363	-0.7806207	0.7517063	1.0405703	0.1618679	-0.9580455	**B_IGKV4-1**	1.5365236	
**MZB1**	-0.5182741	-0.8193798	0.2709697	0.4202210	0.3025775	-0.9903766	**B_MZB1**	-0.8895748	
**NR3C2**	-0.9253023	-0.3058538	0.2362751	0.2609131	0.1533608	0.0454577	**B_NR3C2**	1.4400662	
**RAB40C**	1.0371634	0.1058014	-0.2067216	-0.0478142	-0.0969775	-0.0191308	**B_RAB40C**	-0.6562120	

*shown are only genes with non-zero elastic net coefficients, i.e. only those which contribute to the prediction of OT

Algorithm for calculating predicted probabilities of operational tolerance (OT):

1. Calculate the unadjusted -ΔCt values as:

$$\mathbf{-\Delta Ct_{\mathit{GENE}}^{\mathit{unadjusted}} = -\,(Ct_{\mathit{GENE}} - Ct_{\mathit{HK}})}$$

where Ct_{GENE} is gene-expression in Ct units for each gene and Ct_{HK} is gene-expression in Ct units for the reference (house-keeping) gene. For NEWELL-g2, *GAPDH* gene was used as a reference. For DANGER-g6, the geometric mean of *ACTB, B2M, GAPDH* and *HPRT1* genes was used as a reference, calculated as:

$$Ct_{HK} = (Ct_{ACTB} + Ct_{B2M} + Ct_{GAPDH} + Ct_{HPRT1})/4$$

2. Code immunosuppressive drug therapy as **0=off** or **1=on** for each of prednisolone (PRED), cyclosporine (CYC), tacrolimus (TAC), azathioprine (AZA) and mycophenolate mofetil (MMF).

3. Calculate the drug-adjusted gene-expression as:

$$\mathbf{-\Delta Ct_{\mathit{GENE}}^{\mathit{drug\text{-}adjusted}} = -\Delta Ct_{\mathit{GENE}}^{\mathit{unadjusted}} - -\Delta Ct_{\mathit{GENE}}^{\mathit{predicted}}}$$

where:

$$\mathbf{-\Delta Ct_{\mathit{GENE}}^{\mathit{predicted}} = A_{DRUGS} + PRED*B_{PRED} + CYC*B_{CYC} + TAC*B_{TAC} + AZA*B_{AZA} + MMF*B_{MMF}}$$

Note that the drug index is 0 when a patient is off a given drug and the corresponding product is also 0 and is thus ignored, e.g. PRED=0 when a patient is off prednisolone and the product $PRED*B_{PRED}=0$. Also note that the signs of the regression coefficients and the intercept (A_{DRUGS}) should be retained as shown in Supplementary Table S3. This means that regression coefficients with negative sign will automatically be subtracted.

4. Calculate the log-odds from elastic net as:

$$\mathbf{\mathit{log\text{-}odds} = A_{ELNET} + \Sigma\,(-\Delta Ct_{\mathit{GENEi}} * B_{\mathit{GENEi}})}$$

where Σ(…) represents the sum of the products of the -ΔCt value and the corresponding elastic net regression coefficient for each gene included in a given signature.

5. Calculate the probability of OT as:

$$\mathbf{\exp(\mathit{log\text{-}odds}) / (1 + \exp(\mathit{log\text{-}odds}))}$$

where exp(…) stands for exponent.

Note 1: To calculate the probability of OT with the drug-adjusted version of a signature use all steps above and the elastic net regression coefficients from column "Drug-adjusted". This means that the value for $-\Delta Ct_{GENE}$ used in Step 4 will be the value for $-\Delta Ct_{GENE}^{drug\text{-}adjusted}$ calculated in Step 3.

Note 2: To calculate the probability of OT with the unadjusted version of a signature omit Steps 2 and 3 and use the elastic net regression coefficients from column "Unadjusted". This means that the value for $-\Delta Ct_{GENE}$ used in Step 4 will be the value for $-\Delta Ct_{GENE}^{unadjusted}$ calculated in Step 1.

Supplementary Figures

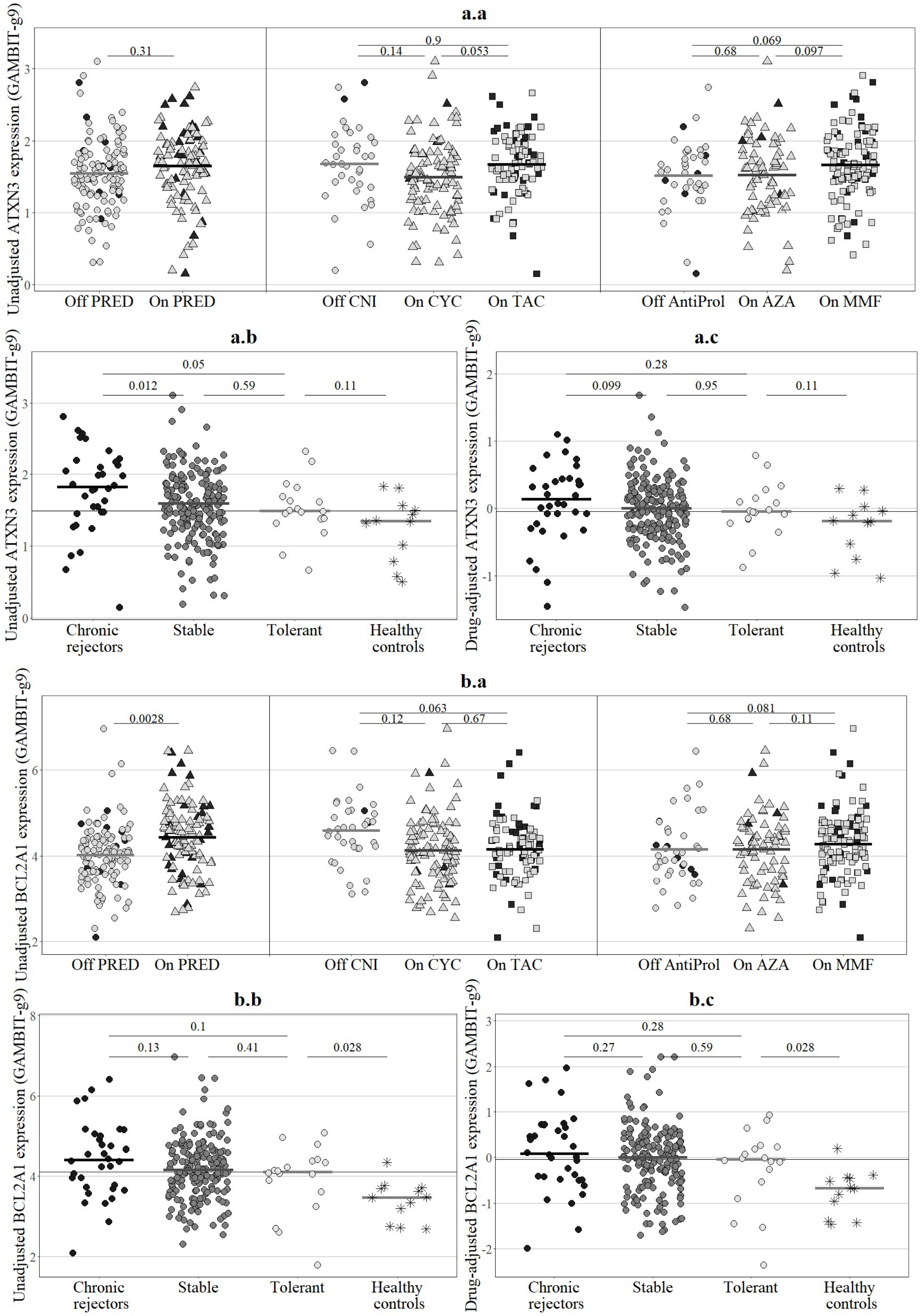

Supplementary Fig. S1a,b Influence of IS drugs on the individual gene-expression levels and the discrimination of operational tolerance *(continues on next page)*

<u>**GAMBIT-g9**</u>**: (a)** ***ATXN3*** gene – R^2 6·0 (3·5–9·5) %; **(b)** ***BCL2A1*** gene – R^2 10·3 (7·4–14·3) %;

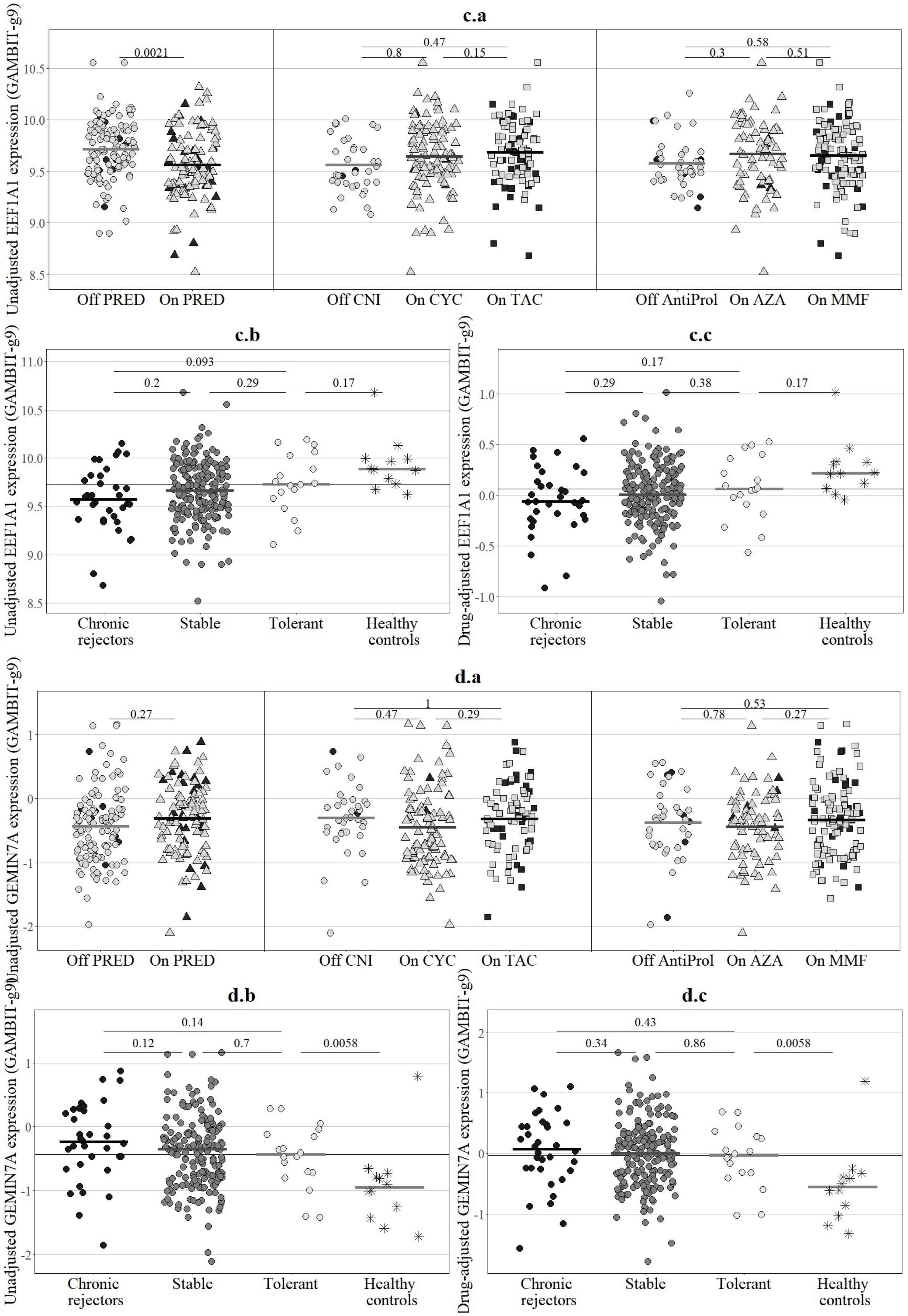

Supplementary Fig. S1c,d Influence of IS drugs on the individual gene-expression levels and the discrimination of operational tolerance *(continues on next page)*

<u>GAMBIT-g9</u>**: (c)** ***EEF1A1*** gene – R^2 7·5 (4·7–10·7) %; **(d)** ***GEMIN7A*** gene – R^2 2·9 (1·2–5·3) %;

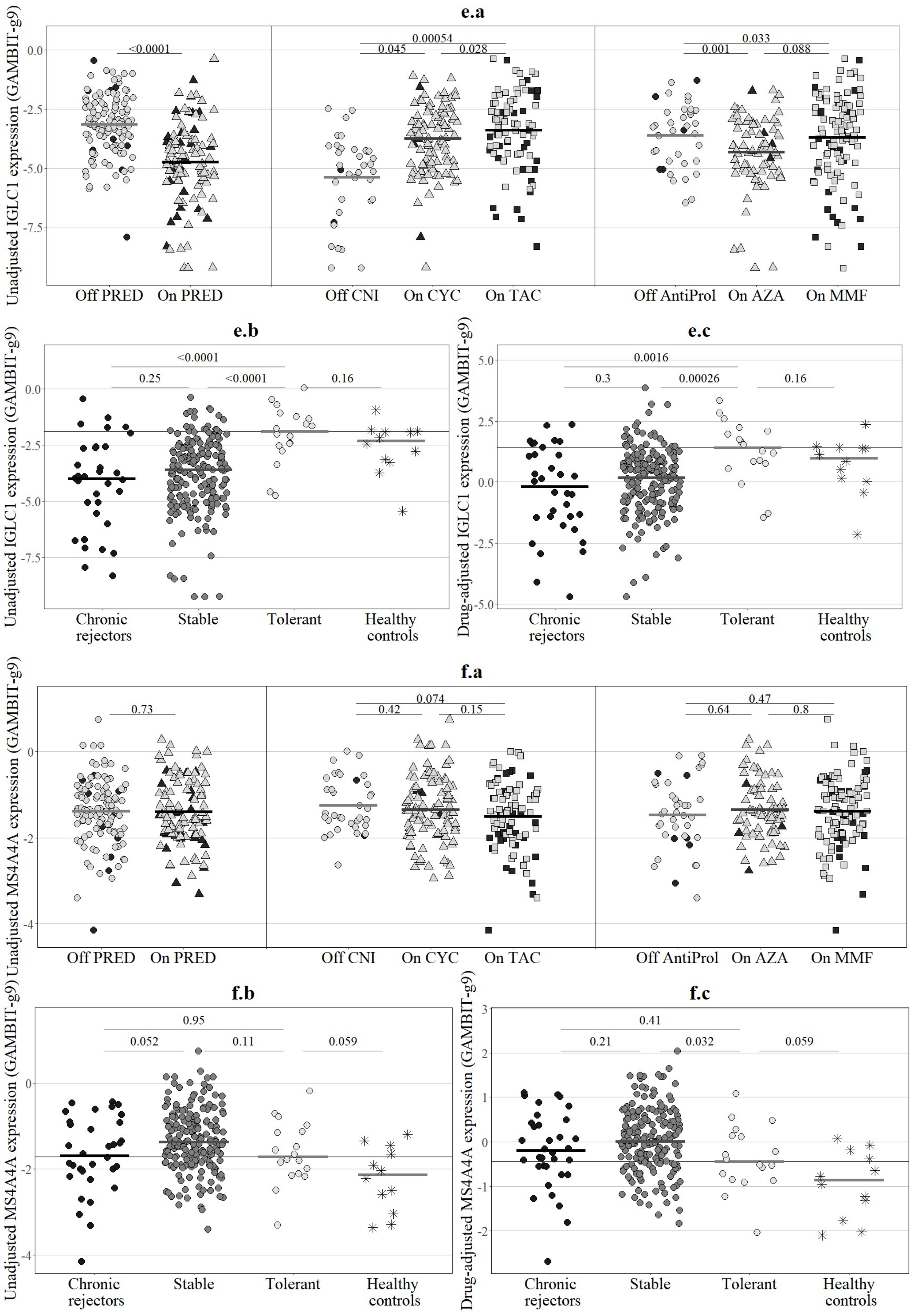

Supplementary Fig. S1e,f Influence of IS drugs on the individual gene-expression levels and the discrimination of operational tolerance *(continues on next page)*

<u>**GAMBIT-g9**</u>**: (e)** ***IGLC1*** gene – R^2 31·9 (27·7-36·2) %; **(f)** ***MS4A4A*** gene – R^2 2·4 (1·0–4·5) %;

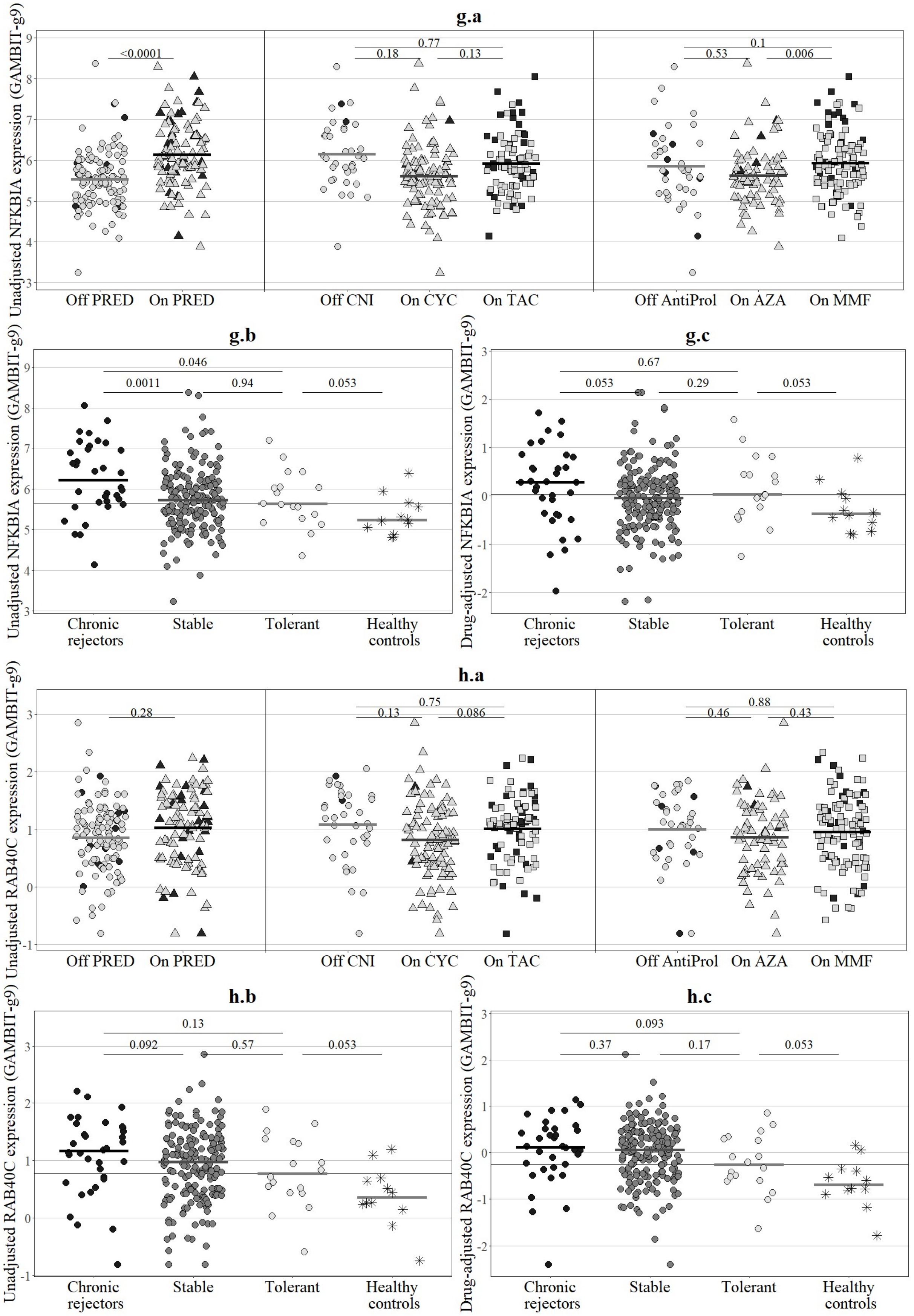

Supplementary Fig. S1g,h Influence of IS drugs on the individual gene-expression levels and the discrimination of operational tolerance *(continues on next page)*

<u>GAMBIT-g9</u>: (g) ***NFKBIA*** gene – R^2 19·3 (15·3–24·8) %; **(h)** ***RAB40C*** gene – R^2 4·5 (2·5–7·7) %;

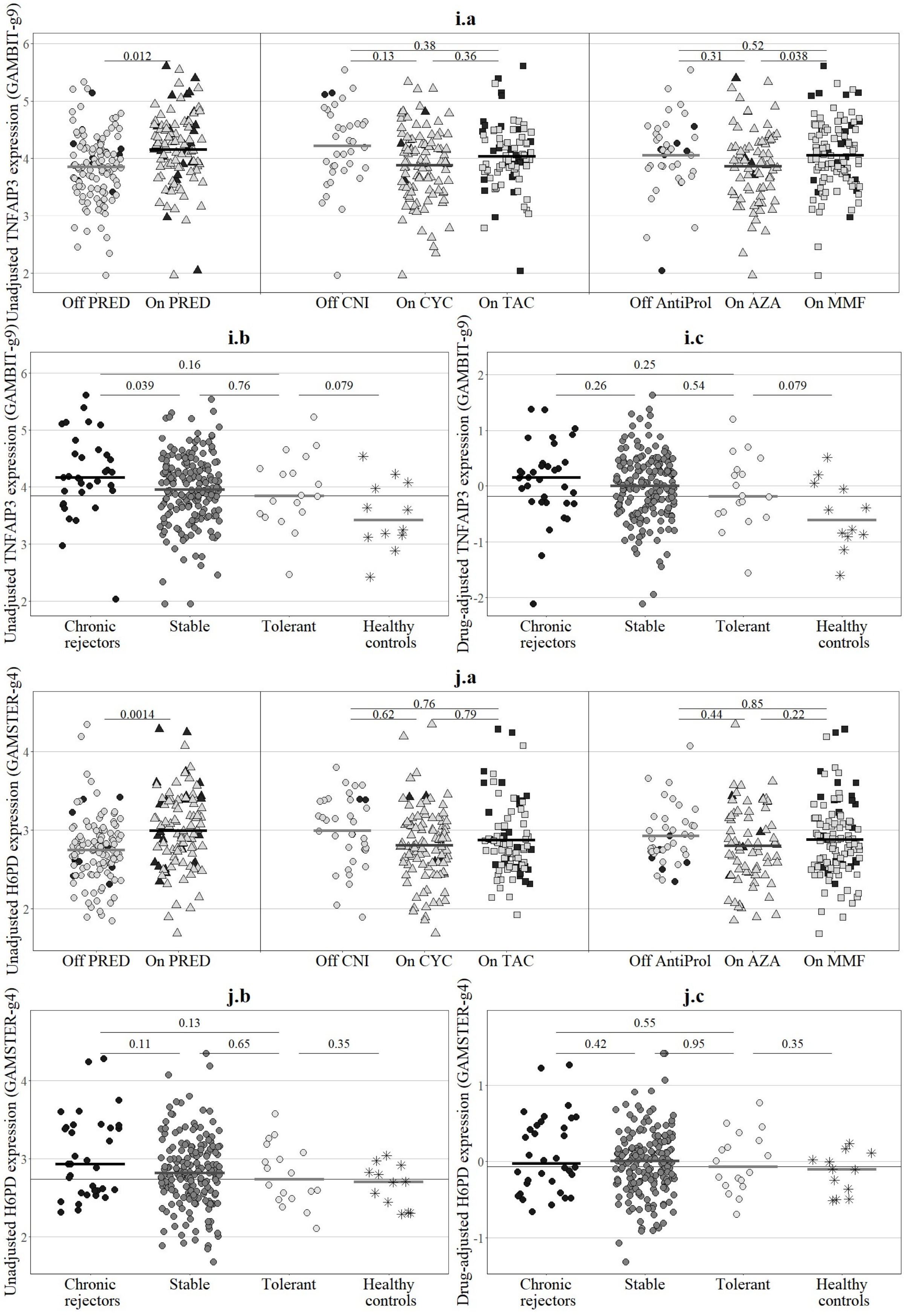

Supplementary Fig. S1i,j Influence of IS drugs on the individual gene-expression levels and the discrimination of operational tolerance *(continues on next page)*

<u>**GAMBIT-g9**</u>**: (i)** ***TNFAIP3*** gene – R^2 8·9 (5·9–13·2) %; <u>**GAMSTER-g4:**</u> **(j)** ***H6PD*** gene – R^2 8·1 (5·4–12·0) %;

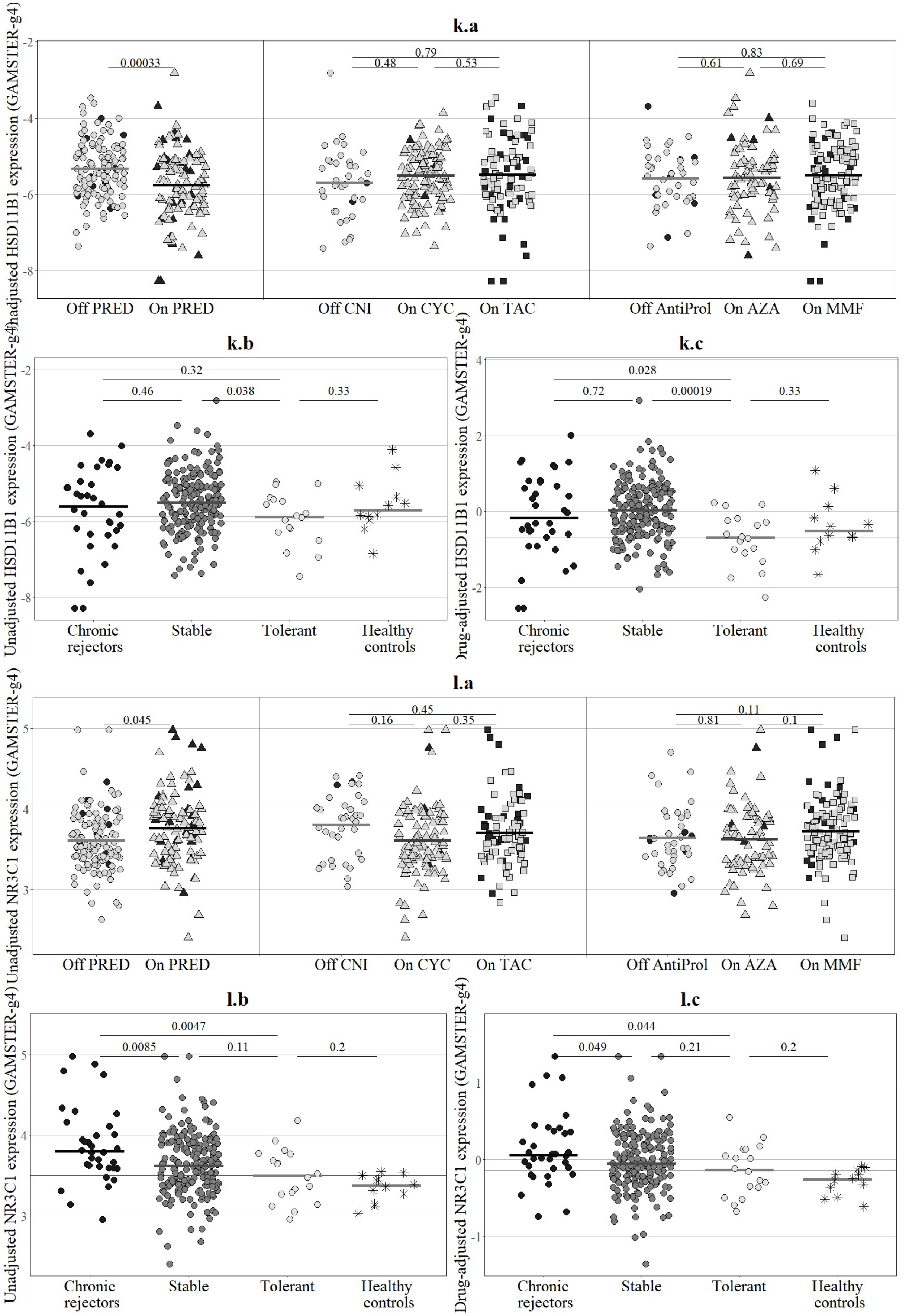

Supplementary Fig. S1k,l Influence of IS drugs on the individual gene-expression levels and the discrimination of operational tolerance *(continues on next page)*

<u>**GAMSTER-g4:**</u> **(k)** ***HSD11B1*** gene – R^2 7·0 (4·8–10·1) %; **(l)** ***NR3C1*** gene – R^2 6·5 (4·1– 9·9) %;

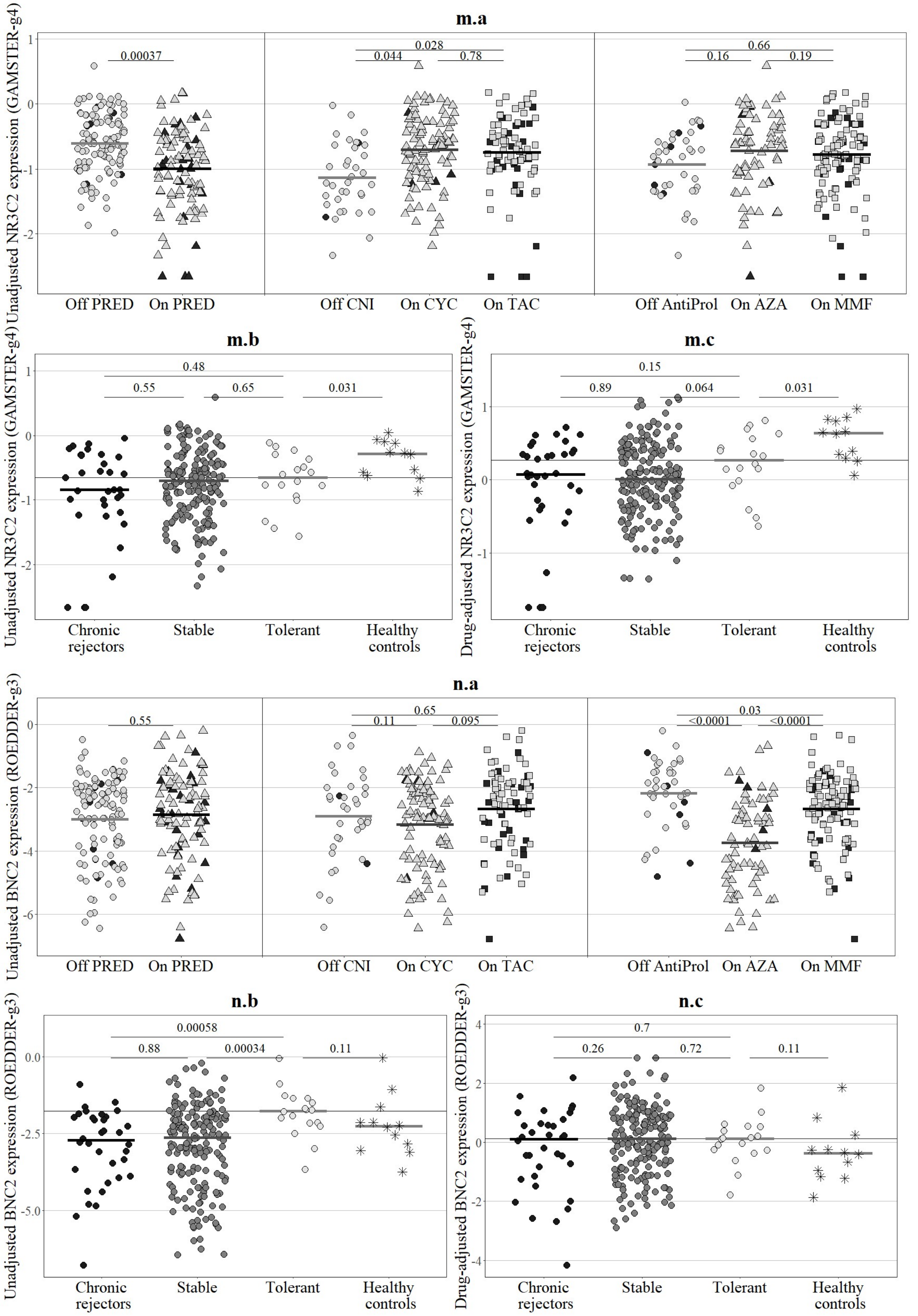

Supplementary Fig. S1m,n Influence of IS drugs on the individual gene-expression levels and the discrimination of operational tolerance *(continues on next page)*

<u>**GAMSTER-g4:**</u> **(m)** ***NR3C2*** gene – R^2 14·8 (11·2–19·0) %; <u>**ROEDDER-g3**</u>: **(n)** ***BNC2*** gene – R^2 20·8 (16·5–25·8) %;

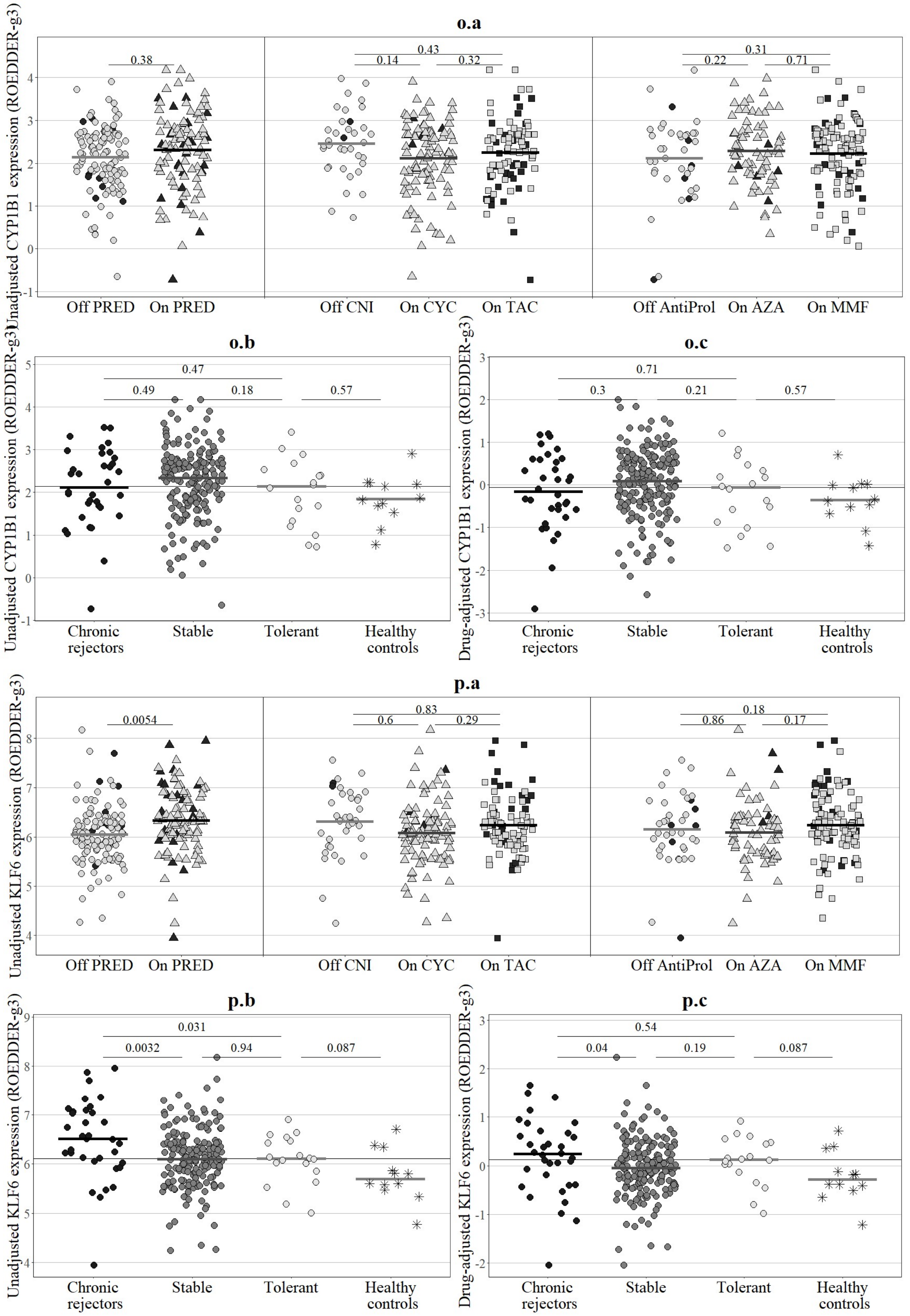

Supplementary Fig. S1o,p Influence of IS drugs on the individual gene-expression levels and the discrimination of operational tolerance *(continues on next page)*

<u>**ROEDDER-g3**</u>: **(o)** ***CYP1B1*** gene – R^2 3·3 (1·4–6·1) %; **(p)** ***KLF6*** gene – R^2 6·9 (4·4–11·1) %;

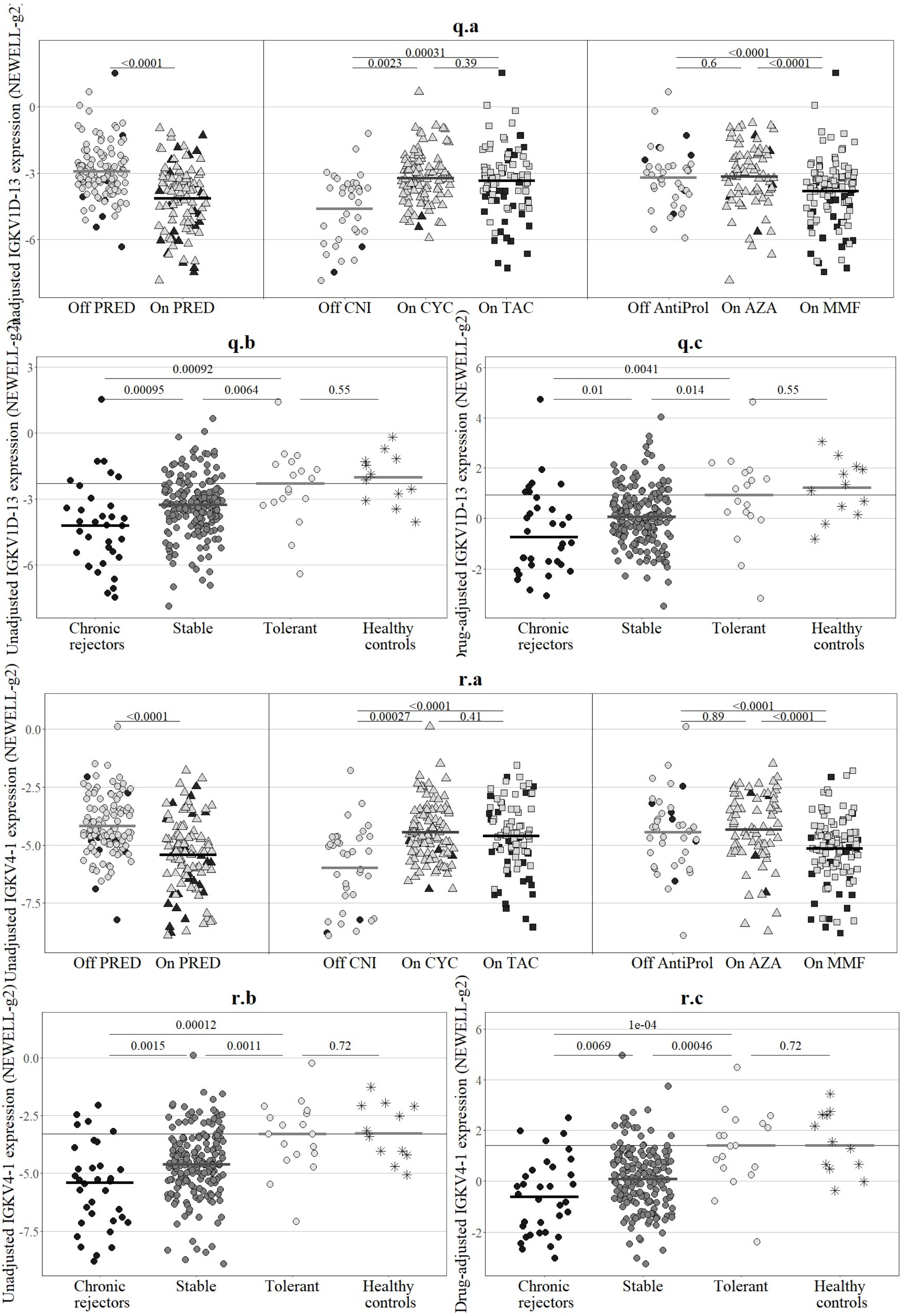

Supplementary Fig. S1q,r Influence of IS drugs on the individual gene-expression levels and the discrimination of operational tolerance *(continues on next page)*

<u>**NEWELL-g2**</u>: **(q)** ***IGKV1D-13*** gene – R^2 28·6 (24·3–34·0) %; **(r)** ***IGKV4-1*** gene – R^2 30·6 (26·4–37·1) %;

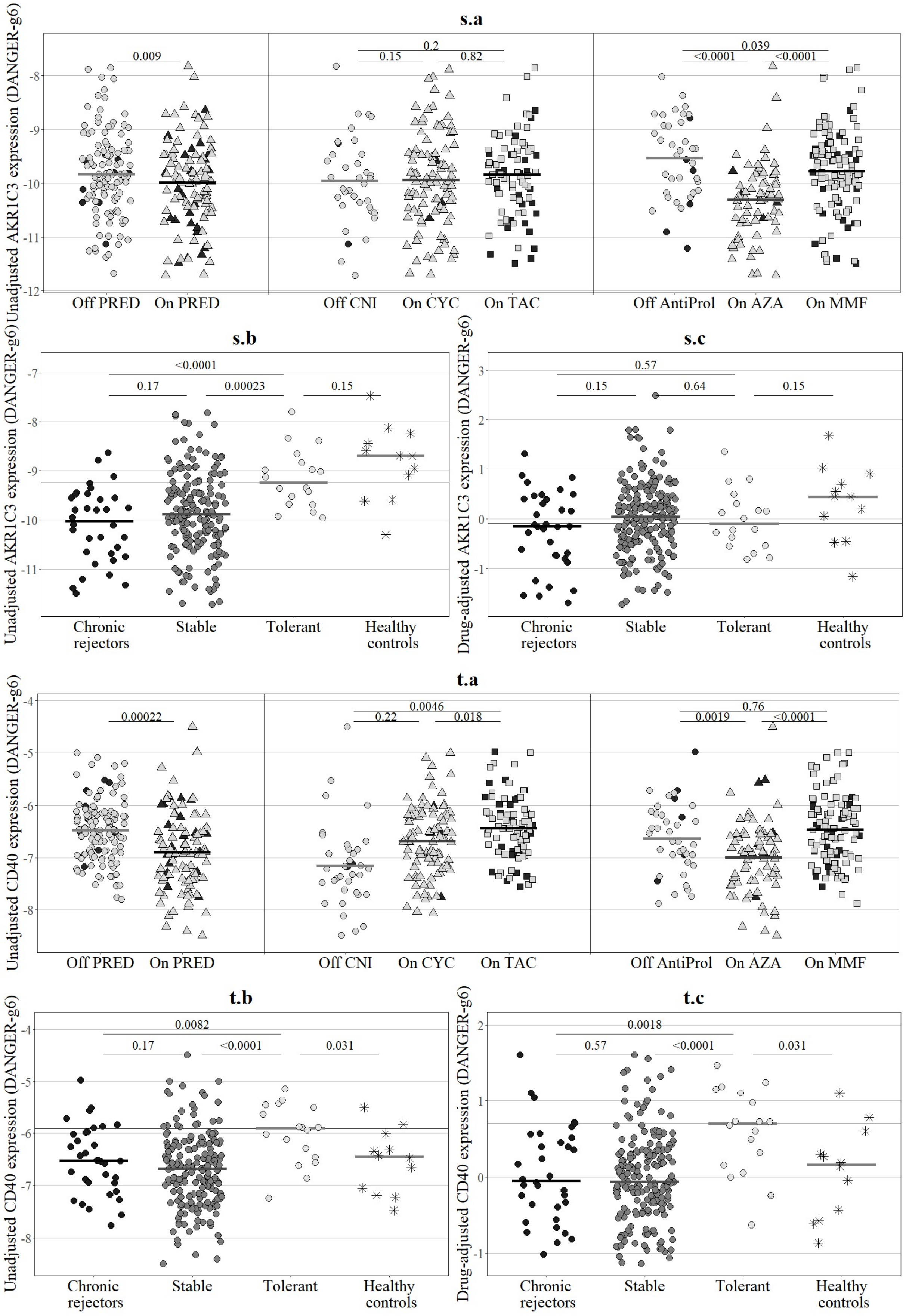

Supplementary Fig. S1s,t Influence of IS drugs on the individual gene-expression levels and the discrimination of operational tolerance *(continues on next page)*

<u>DANGER-g6</u>: **(s)** ***AKR1C3*** gene – R^2 16·6 (13·3–21·1) %; **(t)** ***CD40*** gene – R^2 23·7 (19·7–31·2) %;

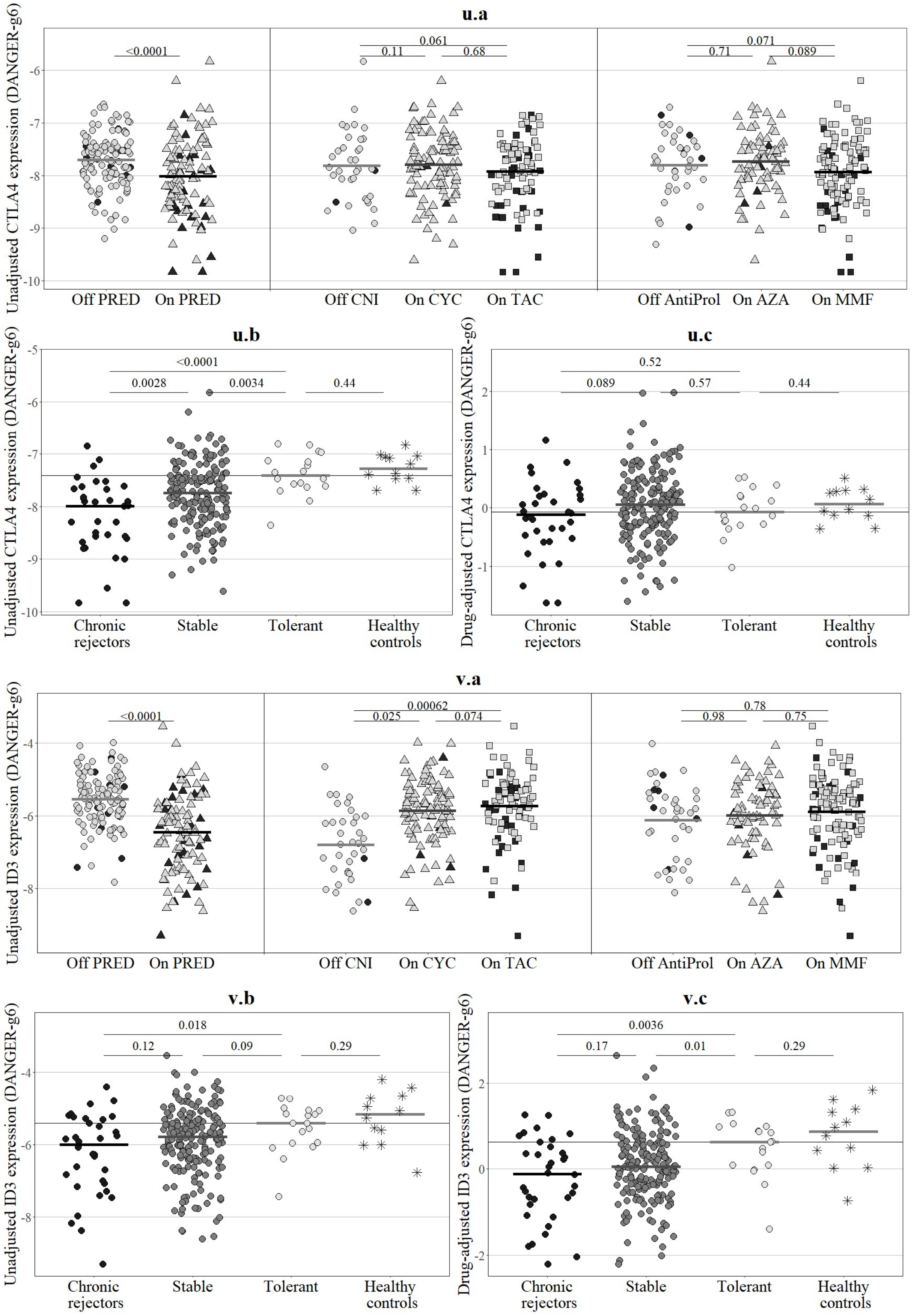

Supplementary Fig. S1u,v Influence of IS drugs on the individual gene-expression levels and the discrimination of operational tolerance *(continues on next page)*

<u>**DANGER-g6**</u>: **(u)** ***CTLA4*** gene – R^2 10·4 (6·9–14·4) %; **(v)** ***ID3*** gene – R^2 25·4 (21·2-30·4) %;

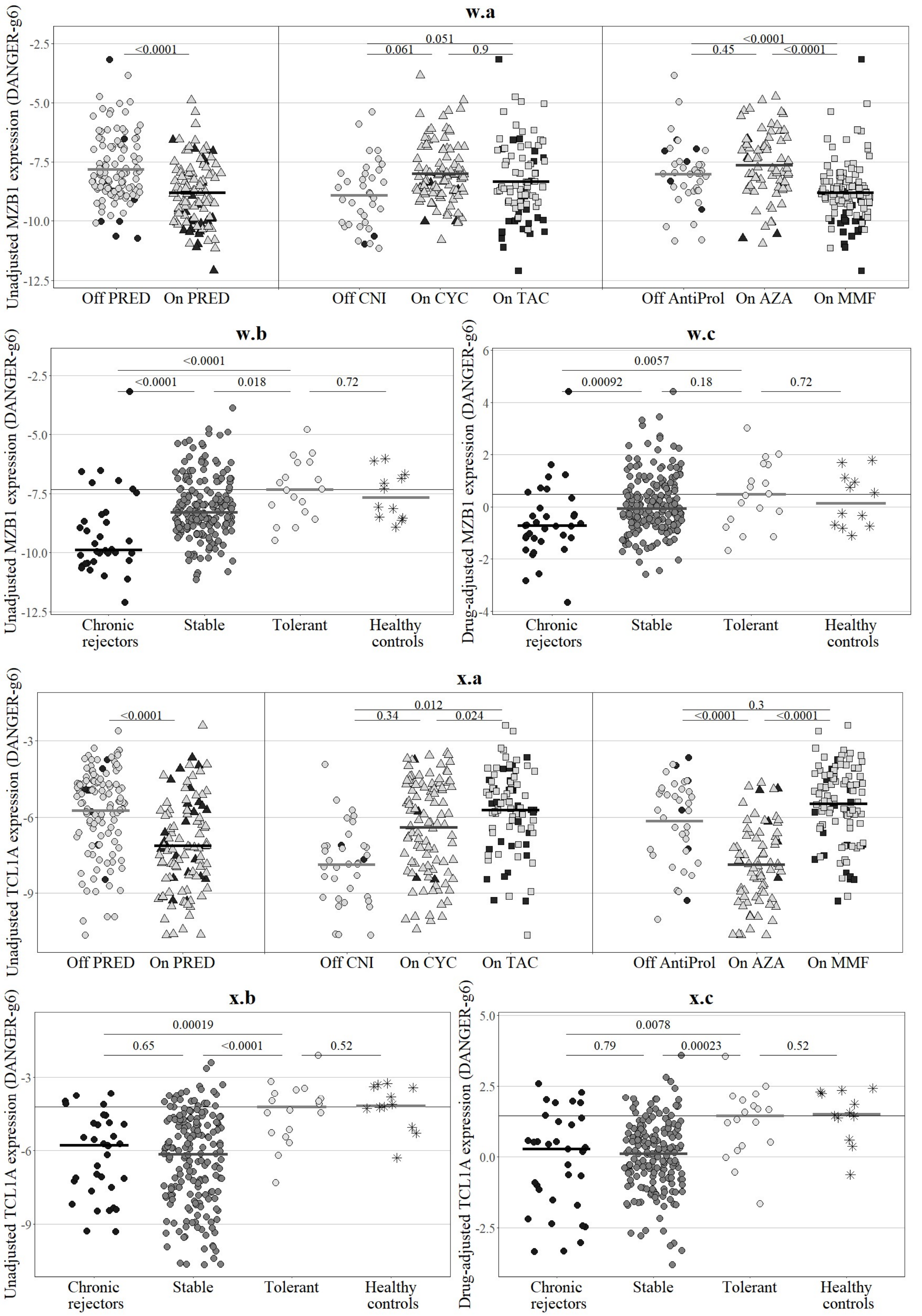

Supplementary Fig. S1w,x Influence of IS drugs on the individual gene-expression levels and the discrimination of operational tolerance *(legend on next page)*

<u>**DANGER-g6**</u>: **(w)** ***MZB1*** gene – R^2 28·6 (24·3–34·4) %; **(x)** ***TCL1A*** gene – R^2 49·9 (46·3–54·0) %;

Supplementary Fig. S1 Influence of IS drugs on the individual gene-expression levels and the discrimination of operational tolerance *(legend)*

Figure panels: **(.a)** – unadjusted gene expression (–ΔCt) with respect to immunosuppressive (IS) drugs; **(.b)** – unadjusted gene expression (–ΔCt) with respect to clinical group; **(.c)** – drug-adjusted gene expression with respect to clinical group; **R^2** – summary of the percentage explained variability from linear models regressing –ΔCt values for gene expression on indicators of IS drug therapy derived during 100 repeats of six-fold cross-validation cycles: mean (2·5th – 97·5th centile range); **Immunosuppressive drugs**: prednisolone (**PRED**) – off/on; calcineurin inhibitors (**CNI**) – off/ on cyclosporine (**CYC**)/ on tacrolimus (**TAC**); anti-proliferative agents (**AP**) – off, on azathioprine (**AZA**)/ on mycophenolate mofetil (**MMF**)); **p-values** (for comparison of groups by drug therapy) – derived from Wald tests in the linear regression models described above, i.e. with adjustment for all other IS drugs (of note, most patients off CNI received prednisolone and *vice versa*, which explains why differences in gene-expression levels observed between off/on CNI are not always matched by low adjusted p-values); **pale symbols** – stable kidney transplant recipients (KTRs); **dark symbols** – KTRs with chronic rejection (CR); **horizontal lines** (for drug therapy groups) – mean gene expression for the group; **gene order** – in alphabetical order per signature, with signatures following the order GAMBIT-g9, GAMSTER-g4, ROEDDER-g3, NEWELL-g2 and DANGER-g6, as defined in Table 1; **p-values** (for comparisons of clinical groups) – derived from Wilcoxon-Mann-Whitney tests, after re-coding of outliers to the next highest/lowest value; **horizontal lines** (for clinical groups) – mean probability of operational tolerance for the group.

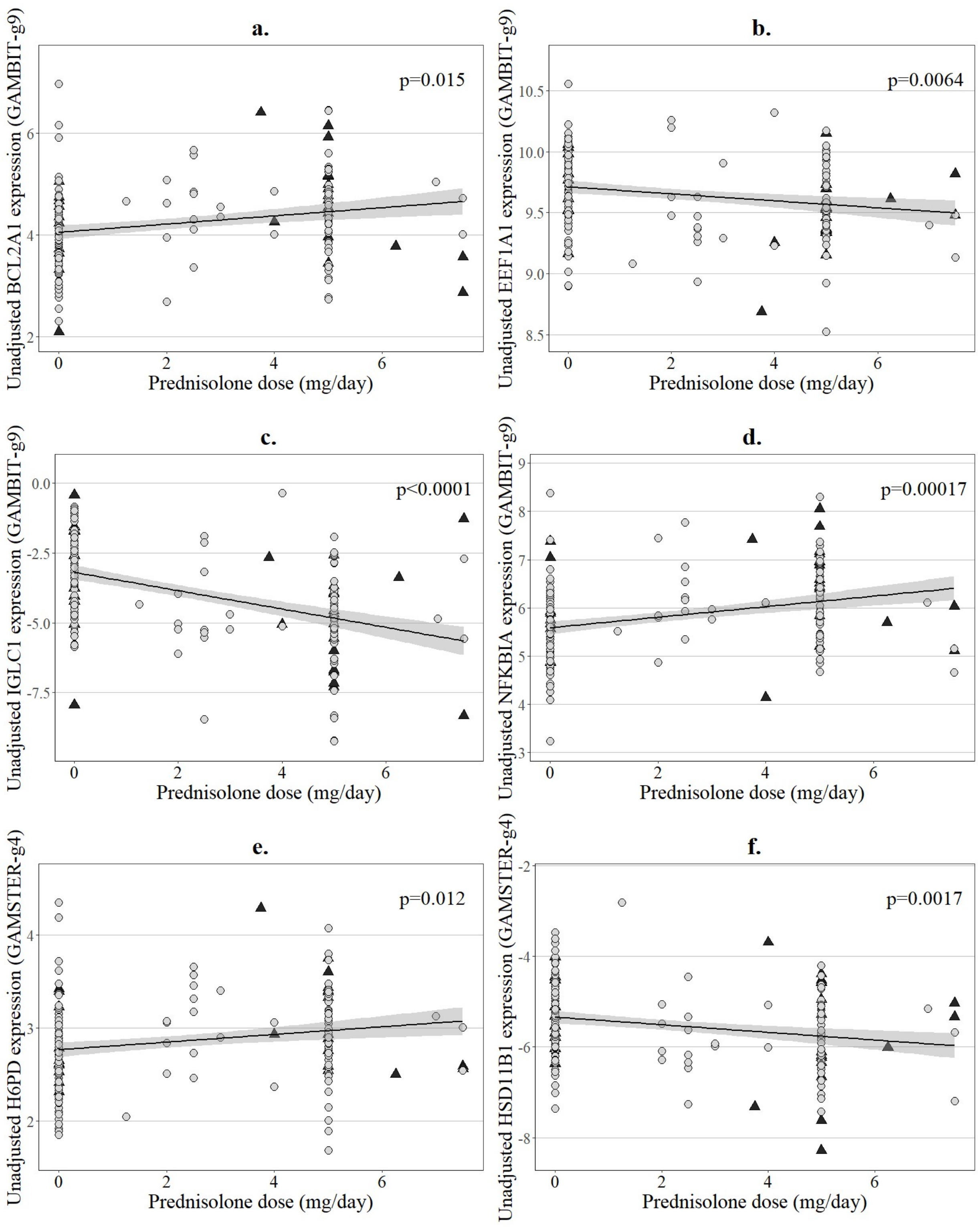

Supplementary Fig. S2a-f Influence of the dose of immunosuppressive drugs on unadjusted gene-expression levels *(continues on next page)*

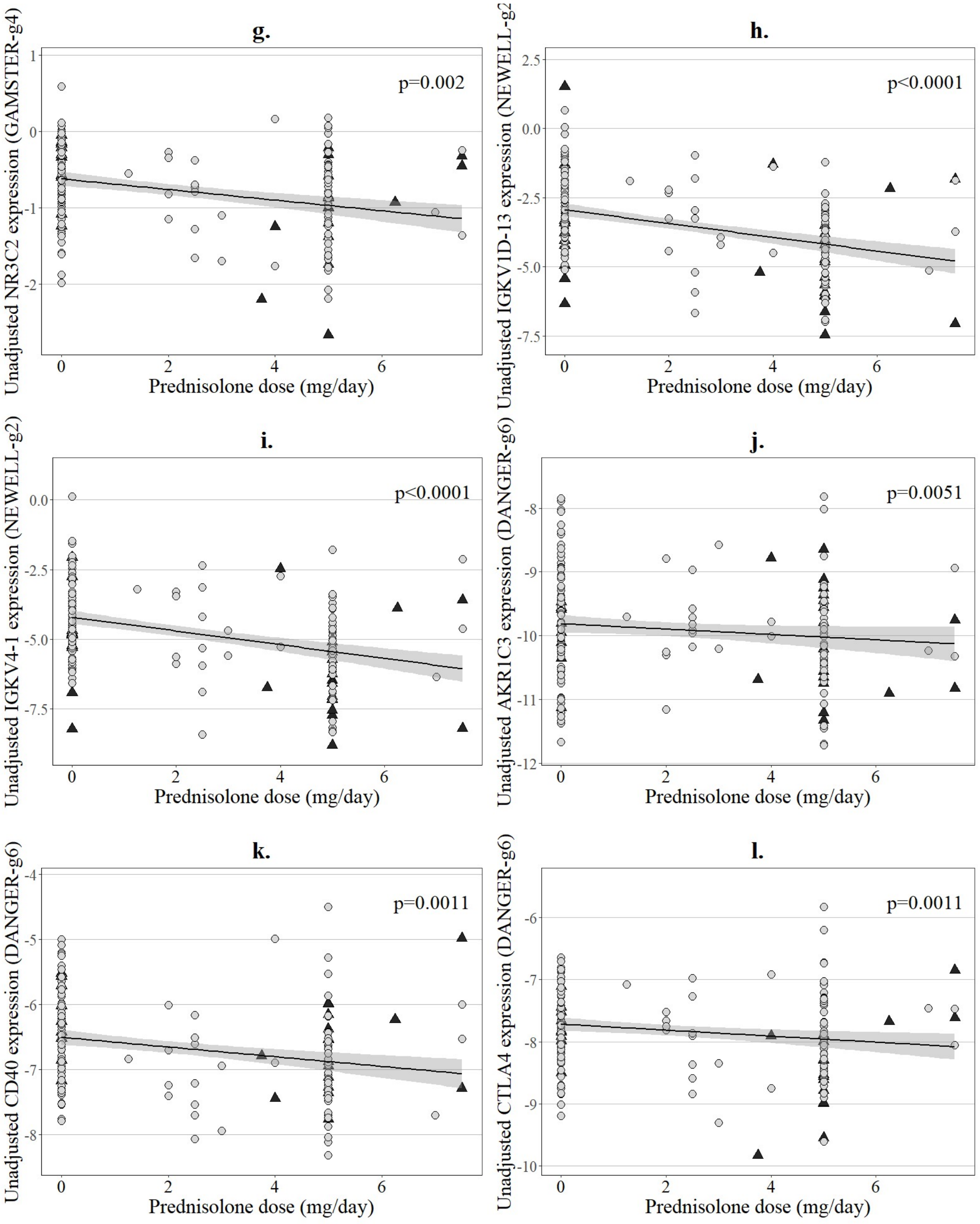

Supplementary Fig. S2g-l Influence of the dose of immunosuppressive drugs on unadjusted gene-expression levels *(continues on next page)*

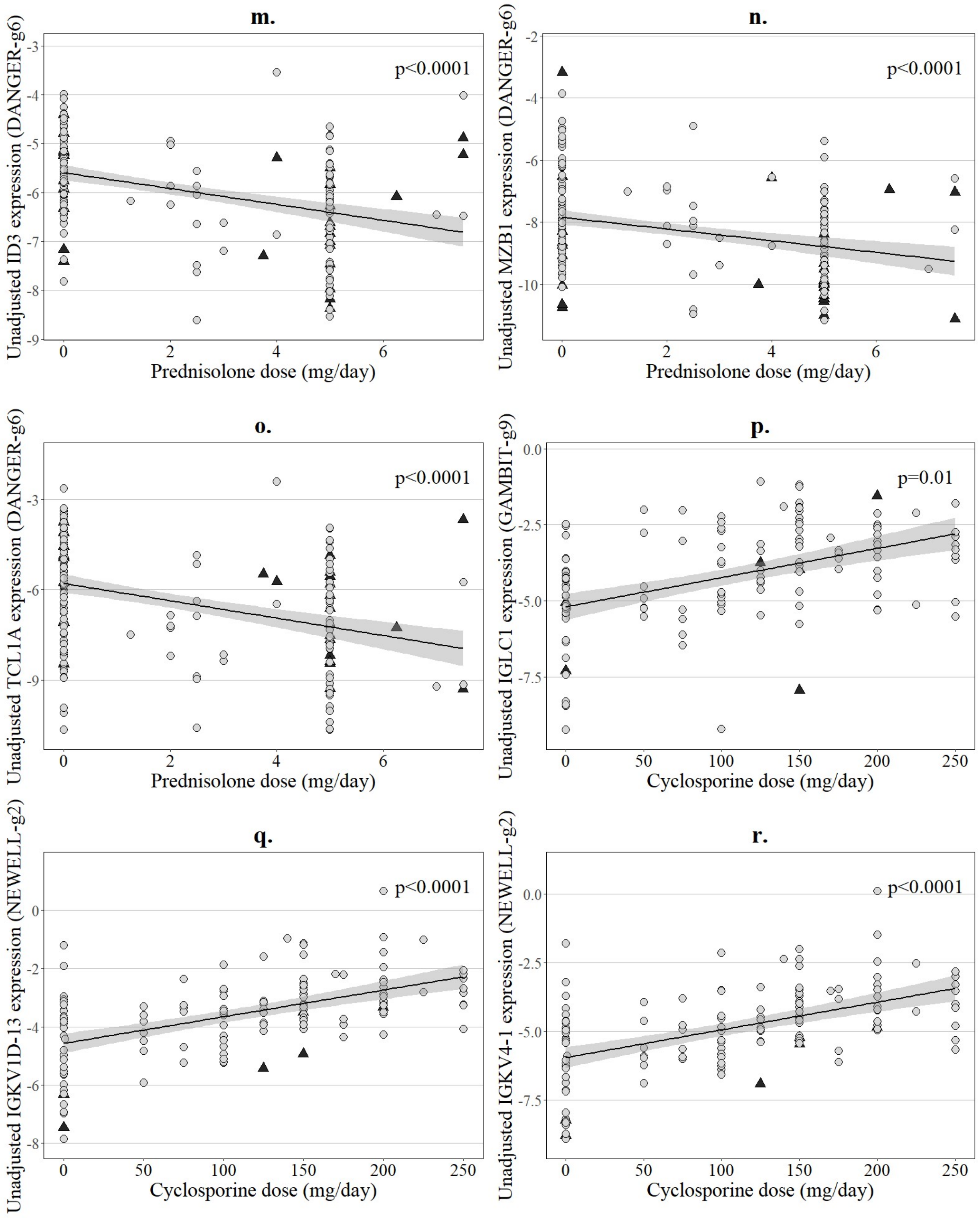

Supplementary Fig. S2m-r Influence of the dose of immunosuppressive drugs on unadjusted gene-expression levels *(continues on next page)*

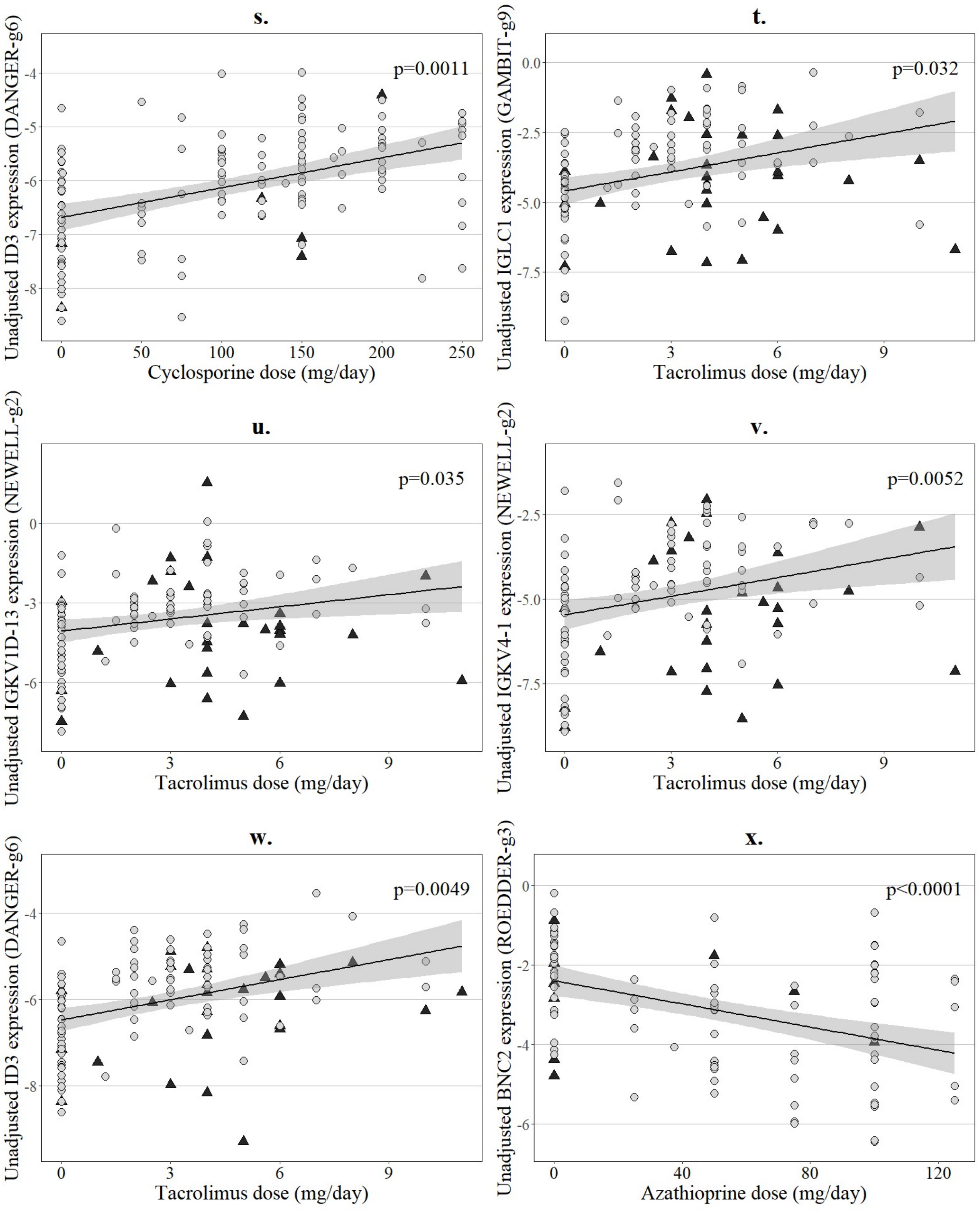

Supplementary Fig. S2s-x Influence of the dose of immunosuppressive drugs on unadjusted gene-expression levels *(continues on next page)*

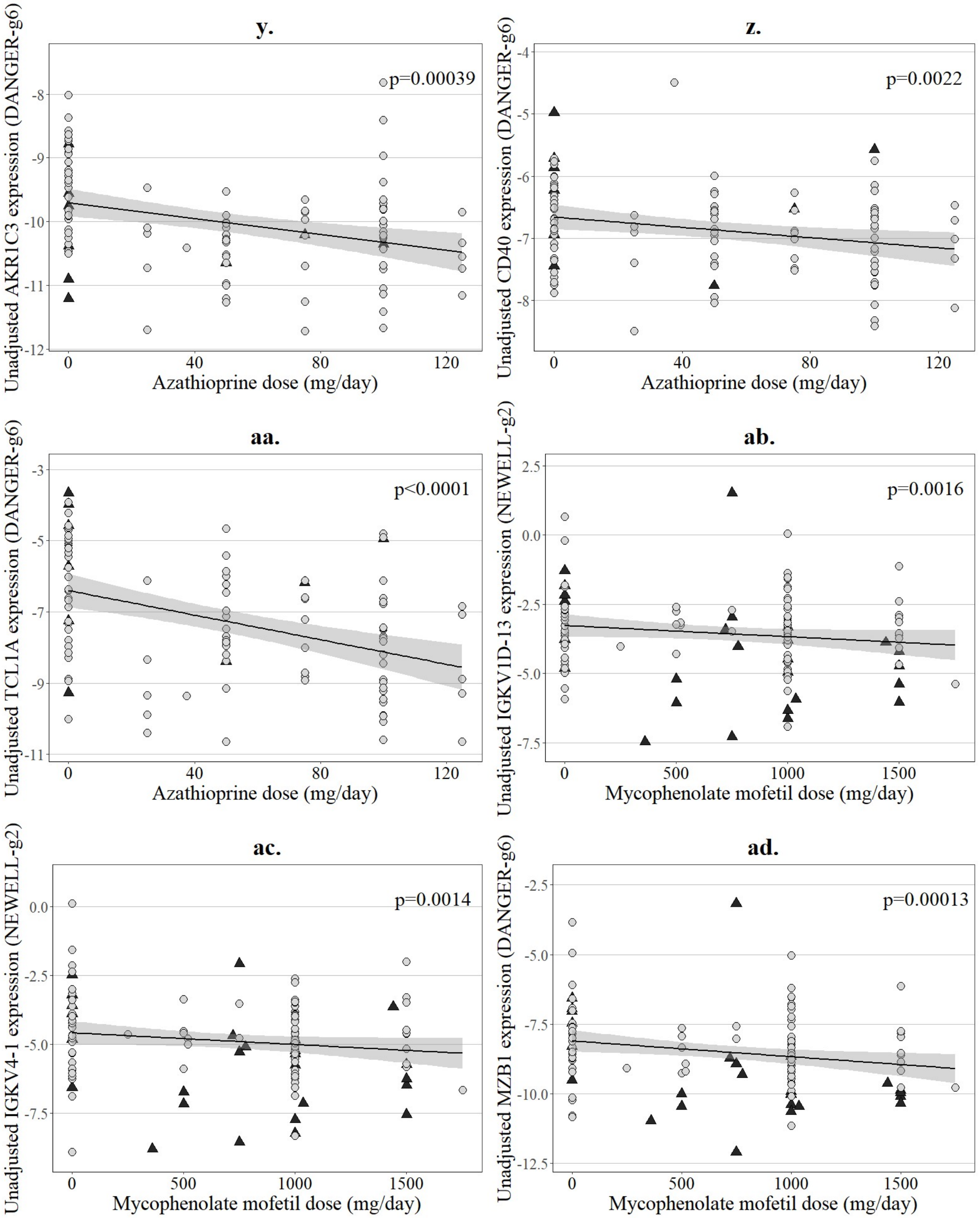

Supplementary Fig. S2y-ad Influence of the dose of immunosuppressive drugs on unadjusted gene-expression levels *(legend)*

Prednisolone: **(a)** ***BCL2A1*** gene from GAMBIT-g9; **(b)** ***EEF1A1*** gene from GAMBIT-g9; **(c)** ***IGLC1*** gene from GAMBIT-g9; **(d)** ***NFKBIA*** gene from GAMBIT-g9; **(e)** ***H6PD*** gene from GAMSTER-g4; **(f)** ***HSD11B1*** gene from GAMSTER-g4; **(g)** ***NR3C2*** gene from GAMSTER-g4; **(h)** ***IGKV1D−13*** gene from NEWELL-g2; **(i)** ***IGKV4−1*** gene from NEWELL-g2; **(j)** ***AKR1C3*** gene from DANGER-g6; **(k)** ***CD40*** gene from DANGER-g6; **(l)** ***CTLA4*** gene from DANGER-g6; **(m)** ***ID3*** gene from DANGER-g6; **n)** ***MZB1*** gene from DANGER-g6; **(o)** ***TCL1A*** gene from DANGER-g6; **Cyclosporine:** **(p)** ***IGLC1*** gene from GAMBIT-g9; **(q)** ***IGKV1D−13*** gene from NEWELL-g2; **(r)** ***IGKV4−1*** gene from NEWELL-g2; **(s)** ***ID3*** gene from DANGER-g6; **Tacrolimus:** **(t)** ***IGLC1***

gene from GAMBIT-g9; **(u)** ***IGKV1D−13*** gene from NEWELL-g2; **(v)** ***IGKV4−1*** gene from NEWELL-g2; **(w)** ***ID3*** gene from DANGER-g6; **Azathioprine:** **(x)** ***BNC2*** gene from ROEDDER−g3; **(y)** ***AKR1C3*** gene from DANGER-g6; **(z)** ***CD40*** gene from DANGER-g6; **(aa)** ***TCL1A*** gene from DANGER-g6; **Mycophenolate mofetil:** **(ab)** ***IGKV1D−13*** gene from NEWELL-g2; **(ac)** ***IGKV4−1*** gene from NEWELL-g2; **(ad)** ***MZB1*** gene from DANGER-g6; **pale symbols** – stable kidney transplant recipients (KTRs); **dark symbols** – KTRs with chronic rejection; **p-values** – derived from Wald tests in linear models regressing unadjusted –ΔCt values for gene expression on IS drug dose, with adjustment for indicators of treatment with all other IS drugs; **regression lines** – linear models including only the drug with examined dose, without adjustment for other IS drugs (the grey bands represent 95% confidence intervals); **exclusions** – in every model were excluded individuals with dose of the examined drug within the top 2.5 centiles; KTRs receiving CYC were excluded from models examining the dose of TAC and *vice versa*, and similarly KTRs receiving AZA were excluded from models examining the dose of MMF and *vice versa*; **gene order** – per drug and in alphabetical order per signature with signatures following the order GAMBIT-g9, GAMSTER-g4, ROEDDER-g3, NEWELL-g2 and DANGER-g6, as defined in Table 1.

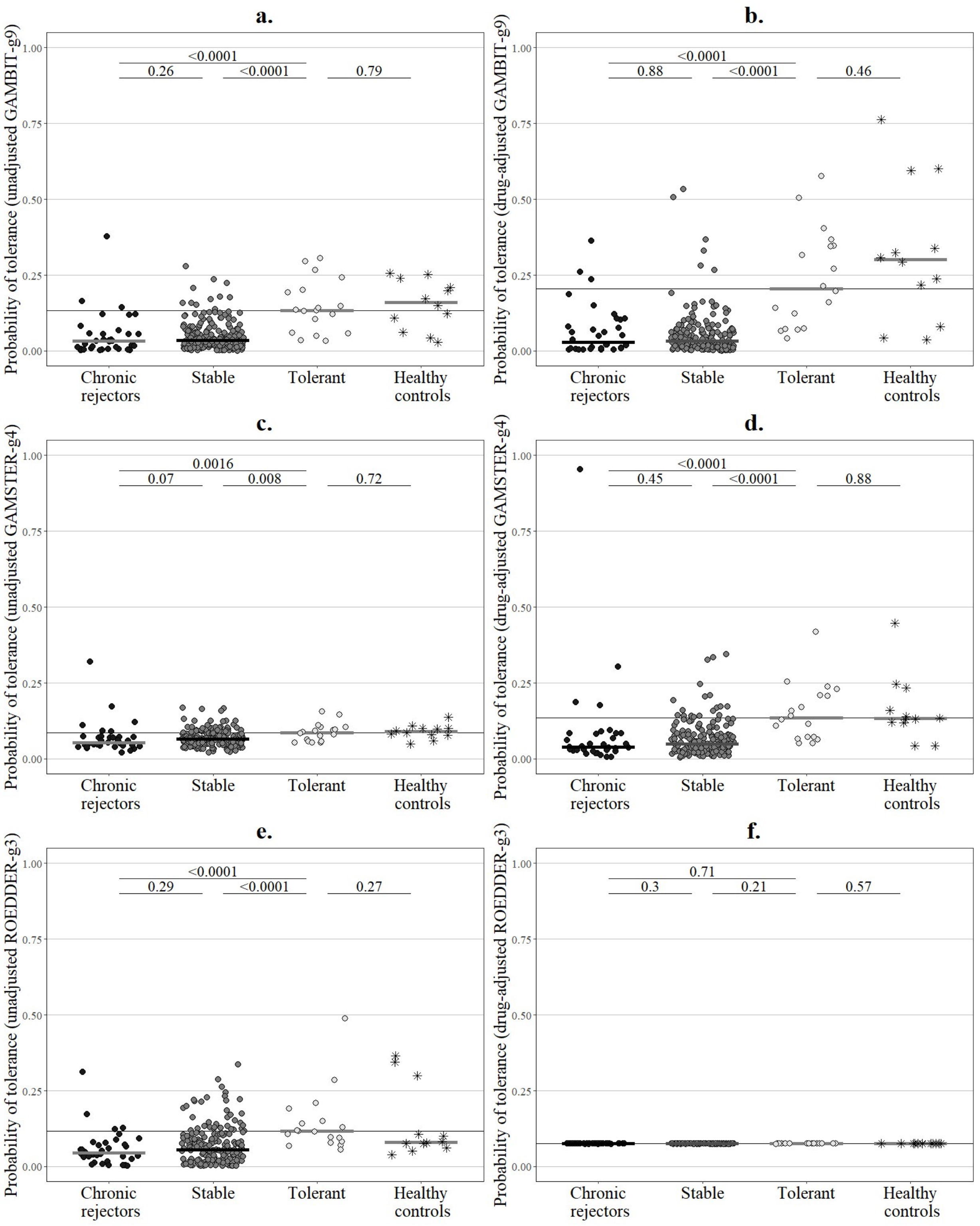

Supplementary Fig. S3a-f Group discrimination achieved by the unadjusted and the drug-adjusted calibration version of gene-expression signatures *(continues on next page)*

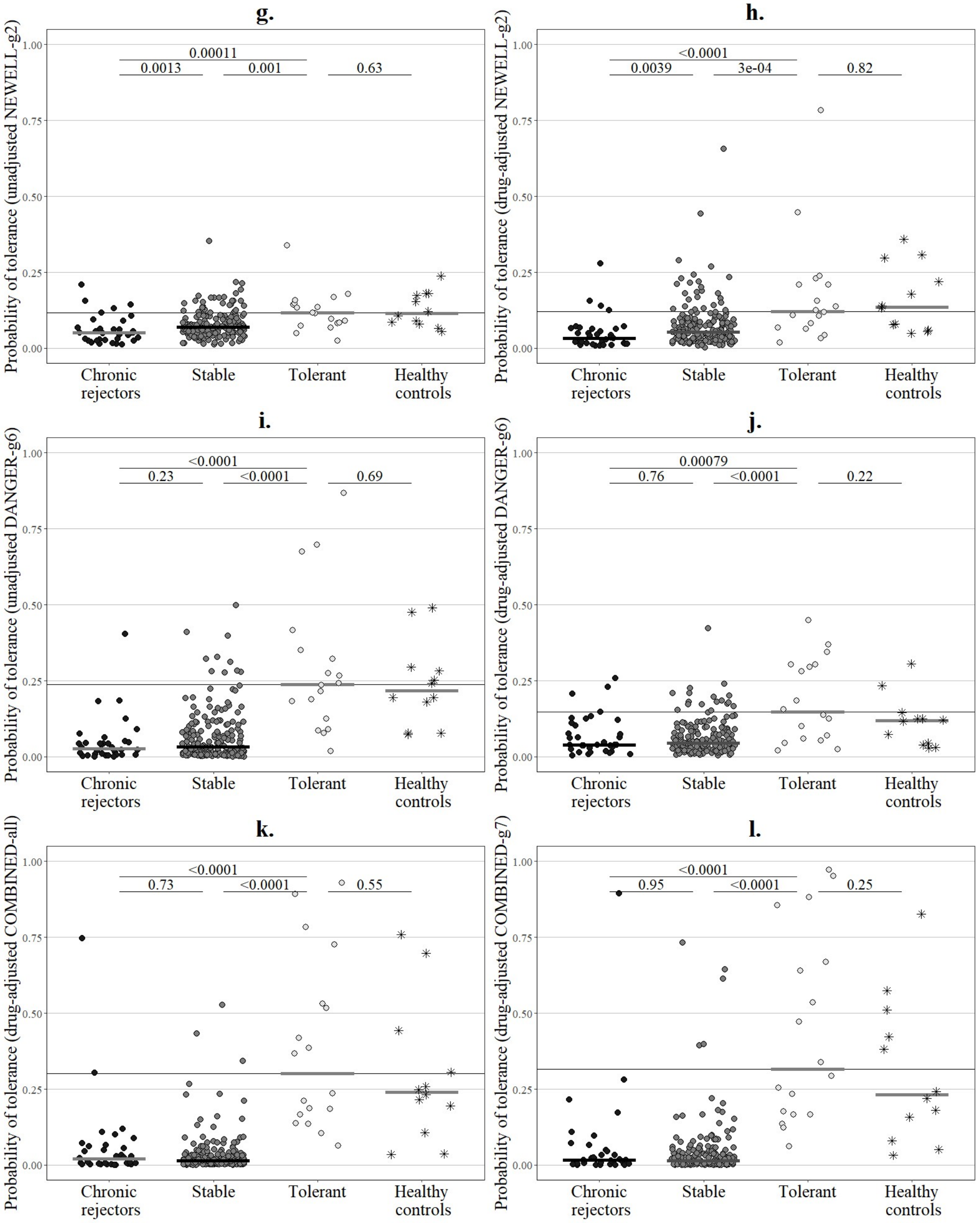

Supplementary Fig. S3g-l Group discrimination achieved by the unadjusted and the drug-adjusted calibration version of gene-expression signatures

(a) GAMBIT-g9 unadjusted; **(b) GAMBIT-g9** drug-adjusted; (c) **GAMSTER-g4** unadjusted; **(d) GAMSTER-g4** drug-adjusted; **(e) ROEDDER-g3** unadjusted; **(f) ROEDDER-g3** drug-adjusted; **(g) NEWELL-g2** unadjusted; **(h) NEWELL-g2** drug-adjusted; **(i) DANGER-g6** unadjusted; **(j) DANGER-g6** drug-adjusted; **(k) COMBINED-all** drug-adjusted; **(l) COMBINED-g7** drug-adjusted; **p-values** – derived from pair-wise Wilcoxon-Mann-Whitney tests after re-coding of outliers to the next highest/lowest value; **horizontal lines for groups** – mean probability of operational tolerance (OT) for the group; **horizontal reference lines** – median probability of OT in tolerant kidney transplant recipients, i.e. a cut-off ensuring 50% sensitivity in the complete T1-cohort.

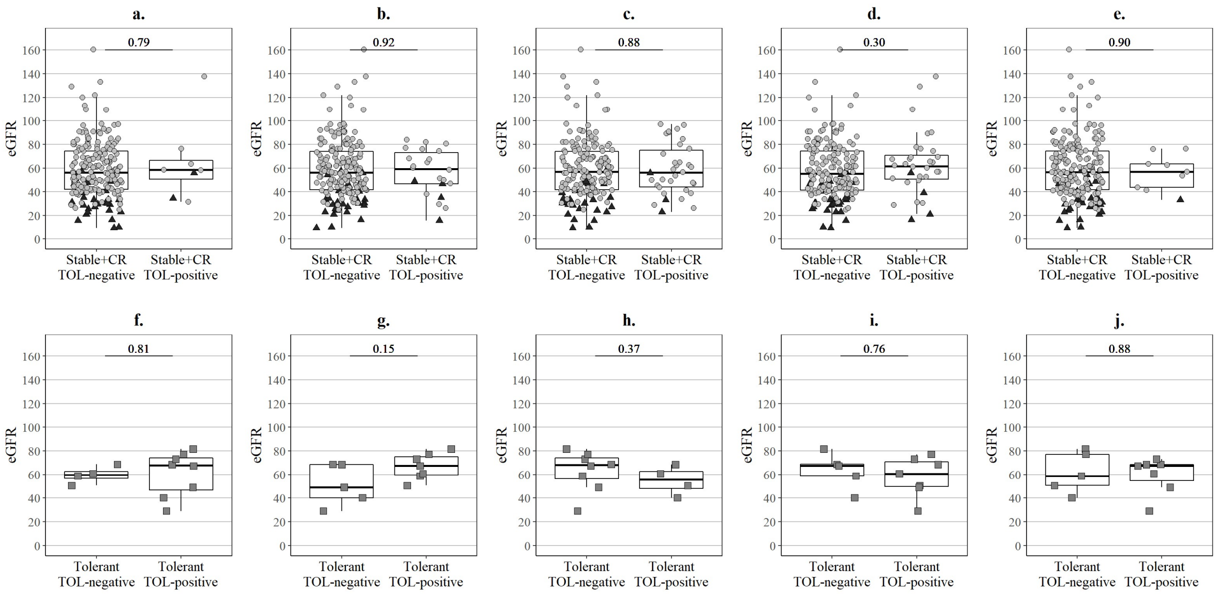

Supplementary Fig. S4 Comparison of estimated glomerular filtration rate (eGFR) between TOL-positive and TOL-negative patients

(a,f) GAMBIT-g9 drug-adjusted; **(b,g) GAMSTER-g4** drug-adjusted; **(c,h) ROEDDER-g3** unadjusted; **(d,i) NEWELL-g2** unadjusted; **(e,j) DANGER-g6** unadjusted; (**a**–**e)** comparison between TOL-positive and TOL-negative non-tolerant kidney transplant recipients (KTRs) (identification as "tolerant" (TOL) by each gene-expression signature was based on using as a cut-off the median of the predicted probabilities of operational tolerance in all tolerant patients from T1-cohort); (**f**–**j)** comparison between TOL-positive and TOL-negative among tolerant KTRs; **grey circles** – stable KTRs; **black triangles** – chronic rejector KTRs; **grey rectangles** – KTRs with operational tolerance; **p-values** – derived from pair-wise Wilcoxon-Mann-Whitney tests. Estimated glomerular filtration rate (eGFR) was available for 12 TOL, 173 stable and 31 CR KTRs from T1-cohort. The signature gene-sets, the reference genes and the drug-adjustment status of gene-expression levels are described in Table 1.

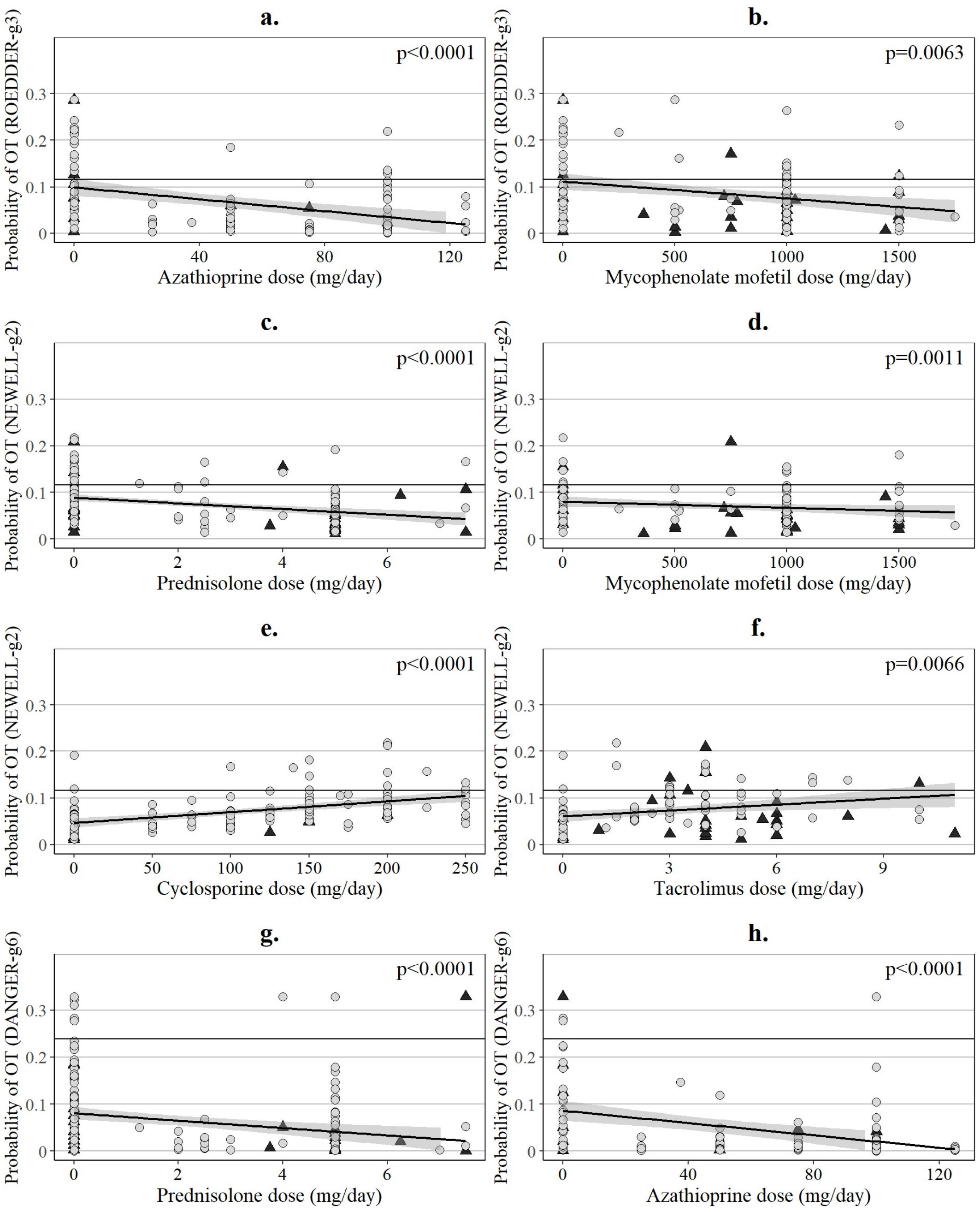

Supplementary Fig. S5. Influence of the dose of immunosuppressive drugs on the predicted probability of tolerance.

(a) ROEDDER-g2 and azathioprine dose; **(b)** ROEDDER-g2 and mycophenolate mofetil dose; **(c)** NEWELL-g2 and prednisolone dose; **(d)** NEWELL-g2 and mycophenolate mofetil dose; **(e)** NEWELL-g2 and cyclosporine dose; **(f)** NEWELL-g2 and tacrolimus dose; **(g)** DANDER-g6 and prednisolone dose; **(h)** DANDER-g6 and azathioprine dose; **pail symbols** – stable kidney transplant recipients (KTRs); **dark symbols** – KTRs with chronic rejection (CR); **p-values** – derived from Wald tests in linear models, based on stable and CR KTRs from T1-cohort, regressing the predicted probability of tolerance transformed to log-odds on the dose of the examined immunosuppressive drug, with adjustment for indicators of treatment with other IS drugs; **horizontal reference lines** – median probability of tolerance in tolerant patients, i.e. a cut-off ensuring 50% sensitivity **regression lines** – linear models including only the drug with examined dose, without adjustment for other IS drugs (the grey bands represent 95% confidence intervals); **exclusions** – in every model were excluded individuals with dose of the examined drug within the top 2.5 centiles; KTRs receiving cyclosporine were excluded from models examining the dose of tacrolimus and *vice versa*, and similarly KTRs receiving azathioprine were excluded from models examining the dose of mycophenolate mofetil and *vice versa*.

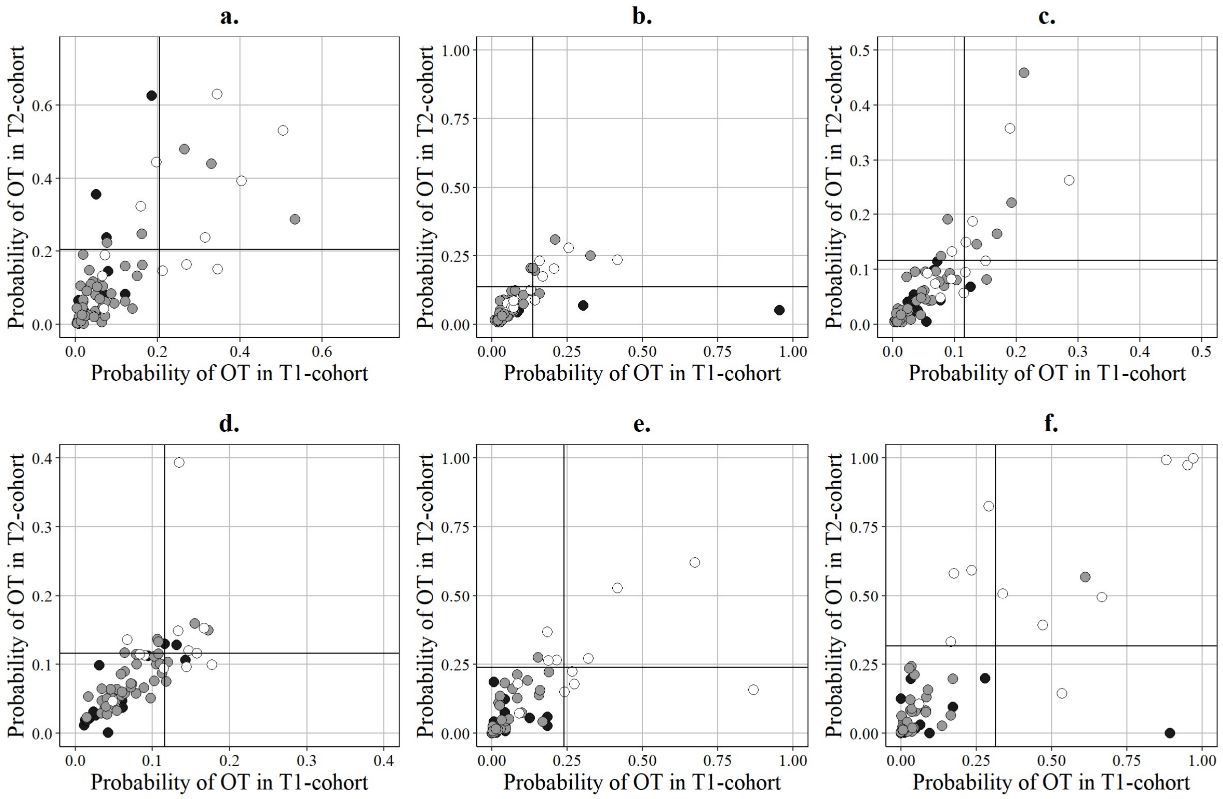

Supplementary Fig. S6 Predicted probability of operational tolerance (OT) for patients with samples collected at both time points 1 and 2

(a) GAMBIT-g9 drug-adjusted; **(b) GAMSTER-g4** drug-adjusted; **(c) ROEDDER-g3** unadjusted; **(d) NEWELL-g2** unadjusted; **(e) DANGER** unadjusted; **(f) COMBINED-g7** drug-adjusted; **T1-cohort** – time point 1 samples from a subset of kidney transplant recipients (KTRs) with samples at both time points; **T2-cohort** – time point 2 samples collected approximately 6 months after the first sample; **black line** – median of the predicted probabilities of tolerance for all KTRs with operational tolerance in the complete T1-cohort; **white circles** – KTRs with operational tolerance; **grey circles** – stable KTRs; **black circles** – chronic rejectors. Signature gene-sets and reference genes are described for each signature in Table 1 Gene-expression is as originally intended for each signature, i.e. unadjusted for ROEDDER-g3, NEWELL-g2 and DANGER-g6 and drug-adjusted for GAMBIT-g9, GAMSTER-g4 and COMBINED-g7. Supplementary Table S2 lists the KTRs identified as TOL-positive by each signature and the immunosuppressive drugs they were treated with.
